# NUMB dysfunction defines a novel mechanism underlying hyperuricemia and gout

**DOI:** 10.1038/s41421-024-00708-6

**Published:** 2024-10-22

**Authors:** Jingwei Chi, Ying Chen, Changgui Li, Shiguo Liu, Kui Che, Zili Kong, Ziheng Guo, Yanchen Chu, Yajing Huang, Libo Yang, Cunwei Sun, Yunyang Wang, Wenshan Lv, Qing Zhang, Hui Guo, Han Zhao, Zhitao Yang, Lili Xu, Ping Wang, Bingzi Dong, Jianxia Hu, Shihai Liu, Fei Wang, Yanyun Zhao, Mengmeng Qi, Yu Xin, Huiqi Nan, Xiangzhong Zhao, Wei Zhang, Min Xiao, Ke Si, Yangang Wang, Yihai Cao

**Affiliations:** 1 Department of Endocrinology and Metabolism, The Affiliated Hospital of Qingdao University, Qingdao, Shandong China; 2 Medical Research Center, The Affiliated Hospital of Qingdao University, Qingdao, Shandong China; 3 Department of Microbiology, Tumor and Cell Biology, Karolinska Institute, Stockholm, Sweden; 4 Institute of Metabolic Diseases, Qingdao University, Qingdao, Shandong China; 5 Department of Medical Genetics, the Affiliated Hospital of Qingdao University, Qingdao, Shandong China; 6 Prenatal Diagnosis Center, the Affiliated Hospital of Qingdao University, Qingdao, Shandong China; 7 Department of Pancreatic Surgery, West China Hospital, Sichuan University, Chengdu, Sichuan China; 8 Department of Spinal Surgery, The Affiliated Hospital of Qingdao University, Qingdao, Shandong China; 9 Department of Endocrinology, The Affiliated Taian City Central Hospital of Qingdao University, Taian, Shandong China; 10 Department of Pathology, The Affiliated Hospital of Qingdao University, Qingdao, Shandong China; 11 Department of Radiology, The Affiliated Hospital of Qingdao University, Qingdao, Shandong China

**Keywords:** Mechanisms of disease, Protein transport

## Abstract

Defective renal excretion and increased production of uric acid engender hyperuricemia that predisposes to gout. However, molecular mechanisms underlying defective uric acid excretion remain largely unknown. Here, we report a rare genetic variant of gout-unprecedented *NUMB* gene within a hereditary human gout family, which was identified by an unbiased genome-wide sequencing approach. This dysfunctional missense variant within the conserved region of the *NUMB* gene (NUMB^R630H^) underwent intracellular redistribution and degradation through an autophagy-dependent mechanism. Mechanistically, we identified the uric acid transporter, ATP Binding Cassette Subfamily G Member 2 (ABCG2), as a novel NUMB-binding protein through its intracellular YxNxxF motif. In polarized renal tubular epithelial cells (RTECs), NUMB promoted ABCG2 trafficking towards the apical plasma membrane. Genetic loss-of-function of NUMB resulted in redistribution of ABCG2 in the basolateral domain and ultimately defective excretion of uric acid. To recapitulate the clinical situation in human gout patients, we generated a NUMB^R630H^ knock-in mouse strain, which showed marked increases of serum urate and decreased uric acid excretion. The NUMB^R630H^ knock-in mice exhibited clinically relevant hyperuricemia. In summary, we have uncovered a novel NUMB-mediated mechanism of uric acid excretion and a functional missense variant of *NUMB* in humans, which causes hyperuricemia and gout.

## Introduction

Gout, as the most common inflammatory arthropathy, continues to be a major health problem around the globe^1^. Gout results from a sustained elevation of serum urate levels (hyperuricemia) that leads to the deposition of monosodium urate crystals in joints, tendons, and other tissues. Typically, gout patients manifest painful and swollen joints engendered by precipitating urate crystals, i.e., urate tophi, which may also occur in the distal fingers and in the axial skeleton. High incidences of gout in certain ethnic groups, such as Taiwanese Aboriginals and Maori, suggest the existence of a genetic link to gout. Although hyperuricemia is necessary for gout development, elevated plasma levels of urate alone may not be sufficient. Other environmental or genetic factors are required for progression from hyperuricemia to gout^2–4^. High prevalence of hyperuricemia and gout is exacerbated by high alcohol consumption, obesity, and hypertension^1,5^.

Complexed mechanisms are entailed in hepatic uric acid production and excretion. As the terminal metabolite of endogenous and exogenous purine, uric acid homeostasis represents the balance between production and complex processes of excretion and reabsorption in the kidney and intestine. More than 70% of uric acid is excreted by kidney and the other is cleared by intestine^6,7^. Thus, studying renal uric acid excretion is crucial in understanding the mechanisms underlying development of hyperuricemia and gout. The reabsorption and secretion of uric acid take place at the discrete segment of the kidney proximal tubule^8,9^. Uric acid transporters in renal tubular epithelial cells (RTECs) are located in various surfaces of these highly polarized cells. The apical urate/anion transporter 1 (URAT1; *SLC22A12* gene)^10^ and basolateral glucose transporter 9 (GLUT9; *SLC2A9* gene)^11,12^ are the key transporters for the uric acid absorption pathway. Excretion of uric acid entails the basolateral urate/dicarboxilate exchangers OAT1 and OAT3^13–15^ and the apical ATP-binding cassette proteins ABCG2^16,17^ and MRP4^18,19^. The ATP-binding cassette G2 (ABCG2) was first discovered as an ATP-dependent multidrug resistant transporter in breast cancer cells^20,21^. Accruing evidence shows that ABCG2 and several other related transporters such as ABCC1 and ABCB1 export a myriad of compounds from cells, including uric acid^16,22^. ABCG2 contains 6 transmembrane domains, an N-terminal single intracellular chain, and a short C-terminal tail, and it forms dimers and even oligomers to become a functional unit^23–25^. Genetic variants of ABCG2 in diverse populations have been linked to high risks of hyperuricemia and gout^16,17,26,27^. Within the gene loci associated with the risk of gout identified, *SLC2A9* and *ABCG2* have the strongest effect on serum urate concentration in the context of complex phenotype loci. In Europeans, the amount of variance in serum urate concentrations explained by *SLC2A9* is 2–3% and *ABCG2* is ~1%^28,29^.

The *NUMB* gene-coded protein NUMB, as a clathrin adapter protein, plays a crucial role in determining the cell fate during development. The protein NUMB is highly conserved from invertebrates to humans and governs asymmetric cell division and specification of various cell types, including neurogenesis and the formation of different neuronal cells^30–33^. NUMB is a natural antagonist for the Notch signaling, which sustains the renewal capacity of stem cells and progenitor cells. Mechanistically, NUMB instigates ubiquitination of Notch1 for proteasomal degradation. In particular, NUMB selectively ubiquinates the membrane Notch1 receptor through interacting with E3 ubiquitin ligase Itch^34^. NUMB also repress Notch1 signaling by promote post-endocytotic sorting of Notch to the late-endosome for degradation^35,36^. Deficiency of NUMB in mice producedes defects in cranial neural tube closure and mice died around embryonic day 11.5, resulting from decreased asymmetric divisions of cortical progenitor cells^37^. Experimental evidence also shows that NUMB is a tumor suppressor by antagonizing the Notch signaling^38^ and by preventing the ubiquitination and degradation of p53^39^. NUMB is classified as a clathrin-sorting protein containing several distinct domains: a phosphotyrosine-binding domain (PTBD), a proline-rich region (PRR), and two carboxyl tripeptide motifs (DPF and NPF). The DPF and NPF motifs entail recruitment of the α-adaptin subunit of adaptor protein complex 2 (AP2) and Eps 15 homology domain-containing proteins (EHDs)^40–42^, whereas the PTBD interacts with the NPXY motif in cargo proteins^43^. NUMB binds to amino acid transporter type 3 (EAAT3, also called SLC1A1) through interacting with the YVNGGF motif in the C-terminus^44^. NUMB regulates EAAT3 endocytosis and polarized sorting to the apical plasma membrane. However, the role of NUMB in facilitating excretion of uric acid is completely unknown.

In this study, we employed various in vitro and in vivo gain- and loss-of-function approaches to provide compelling evidence that NUMB physically interacted with ABCG2 and regulated its surface distribution. A natural missense variant, NUMB^R630H^, was identified in a large family with hereditary hyperuricemia and gout. NUMB^R630H^ knock-in in mice reproduced hyperuricemia, inflammatory arthropathy, and kidney impairment phenotypes as seen in human patients. On the basis of these findings, we propose that normalization of NUMB function provides an important therapeutic approach for treating hyperuricemia and gout.

## Results

### Identification of a genetic variant of NUMB in patients with inherent hyperuricemia and gout

**Fig. 1:**
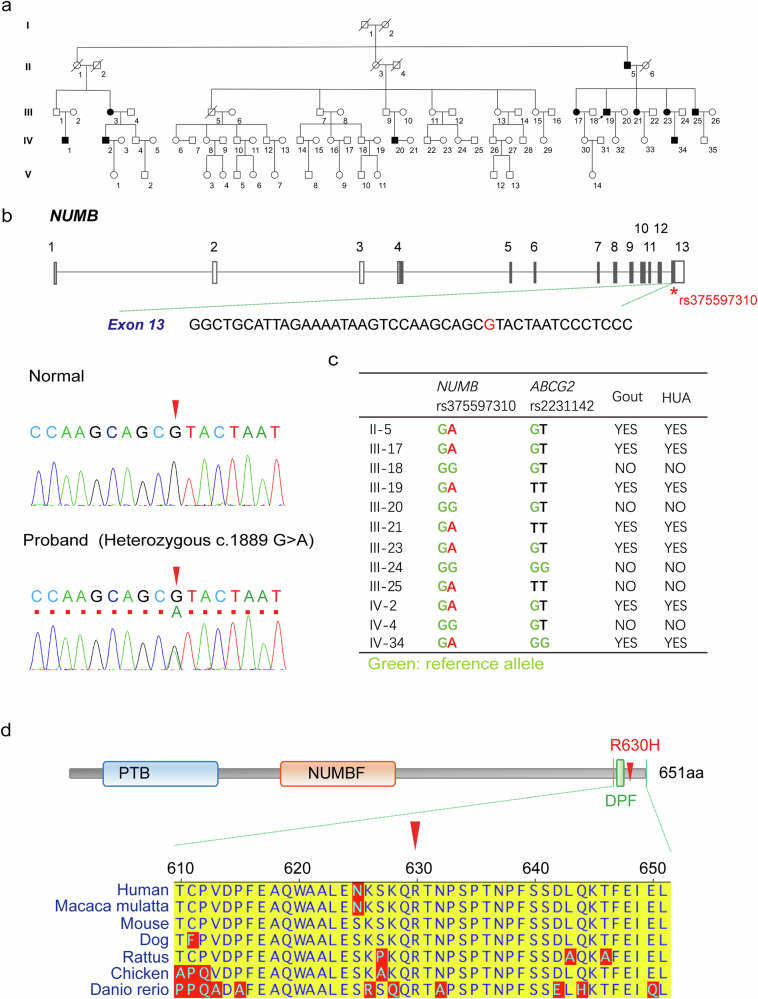
Genetic alteration of *NUMB* gene in patients with inherent hyperuricemia and gout. **a** The pedigree of family with inherent hyperuricemia and gout. The squares and the circles indicate males and females, respectively. The diagonal line indicates an individual who had died. Patients with both hyperuricemia and gout are indicated with black-filled shapes. The arrow indicates proband. **b** *NUMB* gene structure. The rectangles indicate exons and the lines indicate introns. The filled rectangles indicate CDS region. *Variant identified in the family with inherent hyperuricemia and gout. Representative electropherograms show that the c.1889 G > A *NUMB* variant (rs375597310) is found in a heterozygous state in the proband (red arrow head, lower plot), but not in the family member who does not have hyperuricemia or gout (red arrow head, upper plot). **c** *NUMB* rs375597310 and *ABCG2* rs2231142 genotype of family members. **d** Distribution of the missense variant (R630H) identified in the family and NUMB protein ortholog alignments. The R630H variant is located at highly conserved residue across species. Red arrow head indicates R630H variant.

We identified a large family in Qingdao, Shandong Province, China, with inherent hyperuricemia and gout. Among the 5 generations of 83 individuals within this family, 11 individuals had gout (Fig. 1a). In the 2^nd^ generation, a germline derived from a male gout patient showed 37% (7 out of 19) of gout patients. All gout patients within the family were diagnosed according to the diagnostic criteria established by the American Society of Rheumatology in 1997, and the secondary gout was excluded. All gout patients and an equivalent number of non-gout individuals received physical, X-ray, and CT examinations. The height, body weight, BMI, fasting blood glucose (FBG), triglyceride (TG), total cholesterol (TC), creatinine (CREA), urea nitrogen (BUN), and serum urate (UA) were measured (Supplementary Table S1). There were several notable clinical characteristics within this family, including: (1) an equal distribution gout incidence between males and females; (2) an early onset of gout in the younger generations with an average onset age of 45-year-old; (3) involvement in multiple joints; and (4) high levels of serum urate. There was no clear correlation between gout and hypertension, hyperlipidemia, cardiovascular disease, and diabetes (Supplementary Table S2).

To define genetic alterations, the genomic DNA samples from 5 patients (II-5, III-17, III-19, III-23, IV-34) and 3 healthy individuals (III-18, III-20, III-24) within the family were subjected for whole-exome sequencing analysis. The following candidate variants were excluded: (1) variants with minor allele frequency (MAF) > 5%; (2) variants within non-coding or splicing region; and (3) synonymous variants. The variants simultaneously satisfy the following criteria were enrolled as the candidates: (1) the maximum allele frequency < 0.005 (1000 G, ESP6500 and gnomAD databases); (2) according to the classification rules of the American College of Medical Genetics and Genomics (ACMG), variants were classified as pathogenic variants, possible pathogenic variants, or variants of uncertain significance. In total, 133 candidate variants were identified, among which 10 candidates were co-segregated with a disease phenotype in an autosomal dominant manner. Using Sanger sequencing, these 10 variants were also applied for detection in another gout individual of distant kinship within the same affected family (IV-2). The two variants, *NUMB* rs374597310 and *BHMT* rs752243322, were identified in the IV-2 patient who lacked the other eight variants, including *SETDB1* rs767499693, *SELENBP1* rs372038412, *SPTA1* rs142379038, *CACNA2D4* rs766444295, *CHST11* rs758987438, *UBE3B* rs934035216, *TMEM120B* rs771974631 and *SLC38A6* rs201457210. According to the above procedure criteria, genetic variants *NUMB* rs374597310 and *BHMT* rs752243322 were identified as the candidates that were likely linked to the onset of gout within the affected family members. None of the variants of the known gout and hyperuricemia-related genes were co-segregated with the disease phenotype in this family, including *ABCG2*, *SLC2A9*, *SLC22A11*, *SLC22A12*, *SLC17A1*, *SLC17A3*, *PDZK1*, and *INHBB*.

**Fig. 2:**
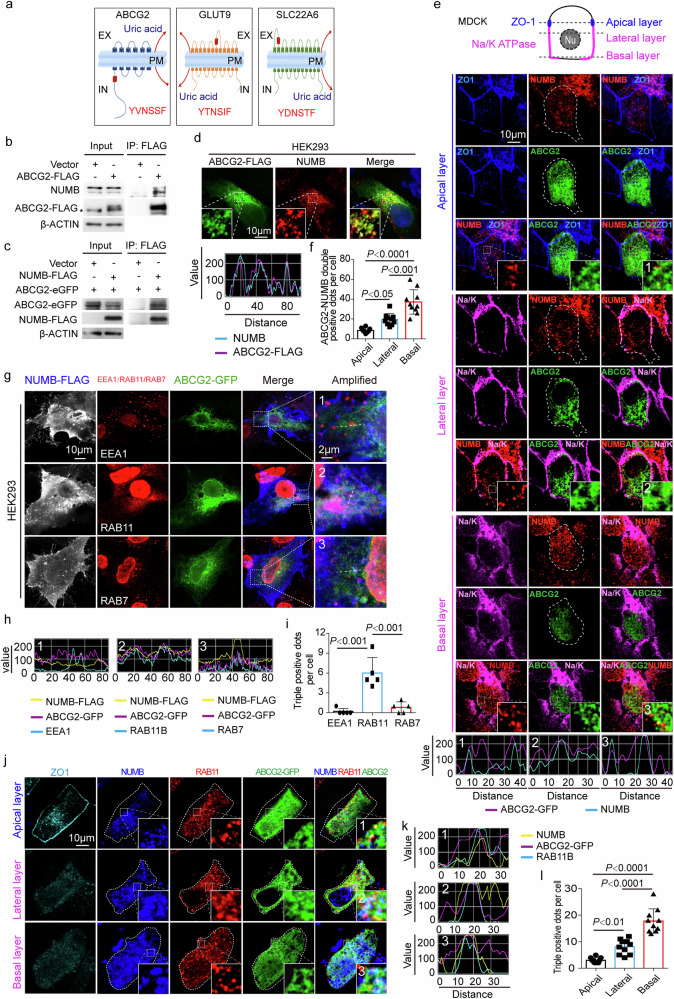
NUMB interacts with ABCG2. **a** Illustration of the three uric acid transporter proteins containing YxNxxF motif. **b**-**c** Co-immunoprecipitation of NUMB and ABCG2. ABCG2-FLAG plasmid or empty vector was transient-transfected into HEK293 cells. Immunoprecipitation was done with FLAG antibody, followed by detecting the co-precipitation of ABCG2-FLAG with endogenous NUMB and ABCG2-FLAG and NUMB in input **b**. Star indicates non-specific band. On the other hand, NUMB-FLAG plasmid or empty vector was co-transfected with ABCG2-eGFP plasmid. Immunoprecipitation was done with FLAG antibody, followed by detecting the co-precipitation of ABCG2-eGFP with NUMB-FLAG and ABCG2-eGFP and NUMB-FLAG in input **c**. **d** Immunofluorescence staining of NUMB and ABCG2 in HEK293 cells. Nuclei were stained with 4′,6-diamidino-2-phenylindole (DAPI). Fluorescent signal intensity along the dashed lines are shown in the bottom panel. **e** Immunofluorescence staining of NUMB and ABCG2 in polarized MDCK cells. ABCG2-eGFP plasmid was transfected into MDCK cells. After reaching 100% confluence, the cells were maintained for another 3–5 days to establish a polarized monolayer. ZO-1 (blue), Na/K ATPase (magenta) and NUMB (red) were stained. NUMB was immuno-stained with rabbit anti-NUMB antibody (Cell signaling technology). Fluorescent signal intensity along lines in the amplified regions numbered with 1–3 are shown in the bottom panel. **f** Comparison of ABCG2-NUMB co-distribution among apical, lateral and basal side with One-way ANOVA with Tukey’s Multiple Comparison Test (*n* = 10 per group). **g** Immunofluorescence staining of NUMB, ABCG2 and early endosome marker EEA1, recycling endosome marker RAB11, late endosome marker RAB7 in HEK293 cells. Fluorescent signal intensity along the dashed lines are shown in **h**. **i** The statistic comparison of the triple positive dots per cells in HEK293 cells were carried out with One-way ANOVA with Tukey’s Multiple Comparison Test (*n* = 5 per group)**. j** Co-distribution of ABCG2-eGFP (green), NUMB (blue) and RAB11 (red) in polarized MDCK cells. ZO-1 (cyan) was stained as an apical marker. **k** NUMB was immuno-stained with mouse anti-NUMB antibody (Santa Cruz). Fluorescent signal intensity along lines in the amplified regions numbered with 1–3 are shown. **l** The statistical comparison of the triple positive dots among in apical, lateral and basal domain were carried out with One-way ANOVA with Tukey’s Multiple Comparison Test (*n* = 10 per group). The results are presented as mean ± SD.

To define *NUMB* rs374597310 and *BHMT* rs752243322 for causing hyperuricemia and gout, we investigated the functions of these two genes. *BHMT* gene encodes a cytosolic enzyme that catalyzes the conversion of betaine and homocysteine to dimethylglycine and methionine. *NUMB* encodes an endocytosis-related protein, which participates in internalization and intracellular trafficking of cargo membrane proteins through binding to motifs of NPxY or YxNxxF (x can be any amino acid) in the cargo proteins^42,44^. Notably, a NUMB-binding motif of YxNxxF was identified in ABCG2, an apical uric acid transporter in polarized RTECs (Fig. 2a). This *NUMB* function encouraged us to investigate its variant role on causing hyperuricemia and gout.

*NUMB* rs374597310 variant was a missense alteration from G to A in the exon 13, position at the coding 1889 (Fig. 1b) with a minor allele frequency of 16/264690 (TOPMED database). This missense variant engendered an amino acid substitution at the position of 630 from arginine (R) to histidine (H), which we defined as NUMB^R630H^ (Fig. 1b). The NUMB^R630H^ variant was located in the C-terminal region of the NUMB protein, which was close to the DPF domain within the highly conserved C-terminus (Fig. 1b, d). Notably, this variant has no clinical significance documented.

The NUMB^R630H^ variant was further validated using the Sanger Sequencing approach, demonstrating heterozygosity within 8 gout patients, but none of healthy family members (Fig. 1b, c). Likewise, the Sanger Sequencing analysis of 100 unrelated healthy individuals did not reveal such a polymorphism. Additionally, we have sequenced 300 sporadic gout patients and did not discover the NUMB^R630H^ polymorphism in this cohort, suggesting a rare genetic variant in human gout patients. These findings demonstrate that NUMB^R630H^ polymorphism exists in gout patients of this genetically inherent family.

### Identification of ABCG2 as a NUMB-binding protein and intracellular colocalization

NUMB-binding motif of YxNxxF identified in the intracellular domain of the N-terminus of ABCG2 (Fig. 2a) suggested a possible physical interaction between ABCG2 and NUMB proteins. Although the YxNxxF motif also exists in other membrane transporters such as GLUT9/SLC2A9 and SLC22A6, those motifs are located in the extracellular domains, excluding interaction with the intracellular NUMB protein (Fig. 2a).

As NUMB consists of four splicing isoforms, we intended to investigate which isoforms were expressed in the kidney. RT-PCR showed a molecular weight corresponding to isoform 2 in human kidney tissues (Supplementary Fig. S1b). Isoforms 1 and 3 were not detectable. We subsequently sequenced the band of isoform 2 and discovered that both isoforms 2 and 4 existed in this band (Supplementary Fig. S1b). These findings indicated that endogenous isoform 2 and perhaps isoform 4 are expressed in the kidney (Supplementary Fig. S1b). Because of the involvement of NUMB isoform 2 in the kidney, we focused on isoform 2 for subsequent gain- and loss-of-function studies.

To study the physical interaction between ABCG2 and NUMB, we performed co-immunoprecipitation experiments, corroborating a physical interaction between NUMB and ABCG2 (Fig. 2b, c). An ABCG2-FLAG protein pulled down the endogenous NUMB protein in HEK293 cells (Fig. 2b). Likewise, a NUMB-FLAG formed a complex with ABCG2 in the co-immunoprecipitation experimental settings (Fig. 2c). Reconciling with immunoprecipitation, ABCG2 and NUMB were largely colocalized in the same subcellular compartments in non-polarized HEK293 cells (Fig. 2d). In MDCK cells, NUMB protein was abundantly accumulated in the basolateral side of polarized MDCK cells. Although NUMB protein was also distributed in the apical side, its positive signals were less robust relative to the basolateral side (Fig. 2e). To further validate these colocalization findings, we employed an independent anti-NUMB antibody, which produced nearly identical distribution patterns (Supplementary Fig. S2). These findings largely reconcile with previously published results^32^. Expectedly, ABCG2 and NUMB were mainly colocalized in the basolateral side of polarized MDCK cells (Fig. 2e, f). Together, these data indicate that ABCG2 physically interacts with NUMB.

NUMB is known to participate in the regulation of membrane protein endocytosis and recycling^41,45^. To study the ABCG2–NUMB complex in the endocytic machinery, we performed colocalization experiments in HEK293 cells using the early endosomal protein marker EEA1, the recycling endosomal marker RAB11, and the late endosomal/lysosomal marker RAB7. Interestingly, co-localization of NUMB and ABCG2 was found predominantly in the RAB11^+^ recycling endosome (Fig. 2g–i). RGB profiling analysis validated the co-distribution of NUMB, ABCG2, and RAB11 positive signals (Fig. 2h). In contrast, the intracellular NUMB and ABCG2 proteins lacked co-distribution with EEA1 and RAB7.

The polarized epithelial cells employed endocytic system to accomplish the directional transport of certain apical membrane proteins^46^. After their synthesis, these proteins are transported to the cell surface, usually the basolateral membrane, from where the proteins are endocytosed and encapsulated within basolateral early endosomes (BEE). The encapsulated proteins were transported to the common recycling endosome (CRE) and subsequently to the apical recycling endosome (ARE), a specialized compartment of polarized epithelial cells^46–48^. NUMB and ABCG2 were colocalized within RAB11^+^ recycling endosomes in polarized MDCK cells. Z-stack imaging analysis demonstrated that NUMB/ABCG2/RAB11 triple positive signals were abundantly expressed in the basolateral side, but rarely in the apical domain of polarized MDCK cells (Fig. 2j–l). These findings suggest that NUMB interacted with ABCG2 within CRE.

### NUMB-mediated relocation of ABCG2 in polarized cells

**Fig. 3:**
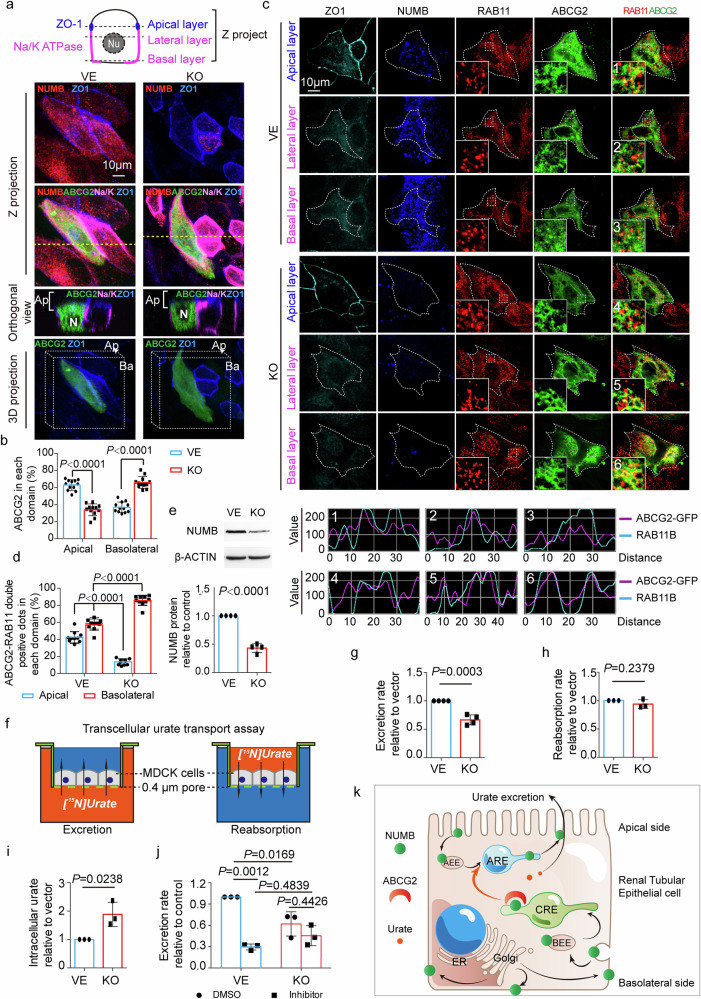
NUMB-knockout abolished the apical distribution of ABCG2 which affects uric acid excretion. **a** Distribution of ABCG2 in polarized *NUMB*-knockout MDCK cells and control cells. ABCG2-eGFP plasmid was transfected into *NUMB*-knockout (KO) or control (VE) MDCK cells. After reaching 100% confluence, the cells were maintained for another 3–5 days to establish a polarized monolayer. ZO-1 (blue), Na/K ATPase (magenta) and NUMB (red) were immuno-stained. The Z projection, orthogonal views and the 3D projection images are shown. N: nuclear; Ap: apical side; Ba: basolateral side. **b** Statistical comparison of the percentage of apical and basolateral ABCG2 between in *NUMB*-knockout and in control MDCK cells (*n* = 12 per group). **c** Co-distribution of ABCG2 (green) and RAB11 (red) in polarized *NUMB*-knockout MDCK cells and control cells. ZO-1 (cyan) was stained as an apical marker. Fluorescent signal intensity along lines in the amplified regions numbered with 1–6 are shown in the bottom panel. **d** Statistical comparison of ABCG2/RAB11-double positive signal in each cellular domain between in *NUMB*-knockout and in control MDCK cells (*n* = 10 per group). **e** Detection of NUMB protein in *NUMB*-knockout (KO) or control (VE) MDCK cells. The representative images of western blotting are shown in the upper panel. β-ACTIN was detected as the loading control. The detection was repeated four times. The statistic result is shown in the bottom panel (*n* = 4 per group). **f** Illustration of trans-cellular uric acid transport assay. **g**–**i** Statistical comparison of the excretion rate (**g**, *n* = 4), reabsorption rate of uric acid (**h,** *n* = 3) and the intracellular uric acid (**i,** *n* = 3) between in *NUMB*-knockout (KO) and in control (VE) MDCK cells. **j** Statistical comparison of uric acid excretion rate among *NUMB*-knockout (KO) and control (VE) MDCK cells treated with ABCG2 inhibitor or solvent DMSO (*n* = 3 per group). **k** Illustration of how NUMB direct ABCG2 to the apical membrane of RTECs. After synthesis, ABCG2 proteins are transported to the cell surface. ABCG2 proteins on the basolateral membrane are endocytosed and encapsulated within BEE, from where the encapsulated ABCG2 were sorted and transported to CRE. NUMB recognizes and binds to ABCG2 in CRE and directs ABCG2 to ARE. From ARE, ABCG2 is delivered to the apical surface. Statistics was calculated using two-tailed unpaired Student’s *t*-test (**e**, **g**–**i**) or Two-way ANOVA with Tukey’s Multiple Comparison Test (**b**, **d**, **j**). The results are presented as mean ± SD.

Having known the physical interaction between NUMB and ABCG2, we studied intracellular distribution of ABCG2 in the presence and absence of NUMB. To achieve this goal, we employed a genetic knockout approach of *NUMB* by the CRISPR-Cas9 gene editing system and achieved over 60% deletion of the NUMB protein (Fig. 3e). In polarized MDCK cells, genetic knockout of *NUMB* resulted in marked redistribution of ABCG2 (Fig. 3a). In the vehicle-transfected control cells, the ABCG2 showed abundant accumulation in the apical membrane (Fig. 3a, b and Supplementary Video S1). By contrast, *NUMB*-knockout abolished the apical distribution of ABCG2, leading to basolateral accumulation of ABCG2 (Fig. 3a, b and Supplementary Video S2). These data demonstrate that NUMB directs ABCG2 to the apical surface of cell membrane in the polarized MDCK cells and disruption of NUMB results in basolateral accumulation of ABCG2.

In non-polarized HEK293 cells, *NUMB* knockout also led to the redistribution of ABCG2. Although *NUMB* knockout had little influence on the total amount of ABCG2 in the plasma membrane (Supplementary Fig. S3b, c), the membrane fraction of ABCG2 showed significant enrichment to the cell–cell contact region in *NUMB* knockout cells (Supplementary Fig. S3a).

Given colocalization of NUMB and ABCG2 within CREs and NUMB guidance of ABCG2 to the apical membrane, we reasonably postulated participation of NUMB in the transcytosis transporting of ABCG2 towards the apical membrane via CRE to ARE. To address this issue, we performed colocalization of ABCG2 and RAB11 in both control and *NUMB*-knockout cells. Interestingly, in *NUMB*-knockout MDCK cells, ABCG2 and RAB11 colocalization in the basolateral side was remarkably increased, whereas their co-distribution in the apical side was reduced (Fig. 3c, d). These findings indicate that NUMB participates in transporting ABCG2 from CREs to AREs.

In non-polarized *NUMB*-knockout HEK293 cells, RAB11 and ABCG2 colocalization was significantly increased, suggesting that accumulation of ABCG2 in the RAB11^+^ recycling endosome in the absence of NUMB. These results suggest that NUMB participates in subcellular distribution of ABCG2 in non-polarized cells (Supplementary Fig. S3e, f).

### Loss-of-function of NUMB affects uric acid excretion in polarized cells

To study the functional impact of NUMB on uric acid excretion and reabsorption, we developed an in vitro cell culture system using the polarized MDCK cells and ^15^N-uric acid. MDCK cells were grown in a nitrocellulose membrane with 0.4-μm pores to confluency to prevent leakiness of small molecules. Addition of the non-membrane permeable small molecules of lucifer yellow dye with molecular weight of 457 Dalton to either upper or lower champers did not detect any leakiness (Supplementary Fig. S4). Addition of ^15^N-uric acid to the lower chamber allowed the measurement of uric acid excretion across the basal membrane to the apical membrane (Fig. 3f). Conversely, incubation of ^15^N-uric acid with MDCK cells in the upper chamber permitted detection of reabsorption of uric acid from the apical membrane to the basal membrane (Fig. 3f).

In the excretion experimental setting, knockout of *NUMB* significantly inhibited ^15^N-uric acid excretion (Fig. 3g). By contrast, reabsorption of ^15^N-uric acid was unaffected by knockout of the *NUMB* gene (Fig. 3h). In concordance with the impaired excretion, the amount of intracellular ^15^N-uric acid was markedly increased in the *NUMB*-knockout MDCK cells (Fig. 3i).

In addition to the genetic approach, we also employed a pharmacological loss-of-function approach using a known ABCG2 inhibitor, Fumitremorgin C. Expectedly, Fumitremorgin C markedly inhibited uric acid excretion in MDCK cells (Fig. 3j). However, Fumitremorgin C-mediated inhibition was debilitated in *NUMB*-knockout cells (Fig. 3j), suggesting that knockout of NUMB sufficiently incapacitates ABCG2 function.

### Intracellular localization and autophagy of NUMB^R630H^

**Fig. 4:**
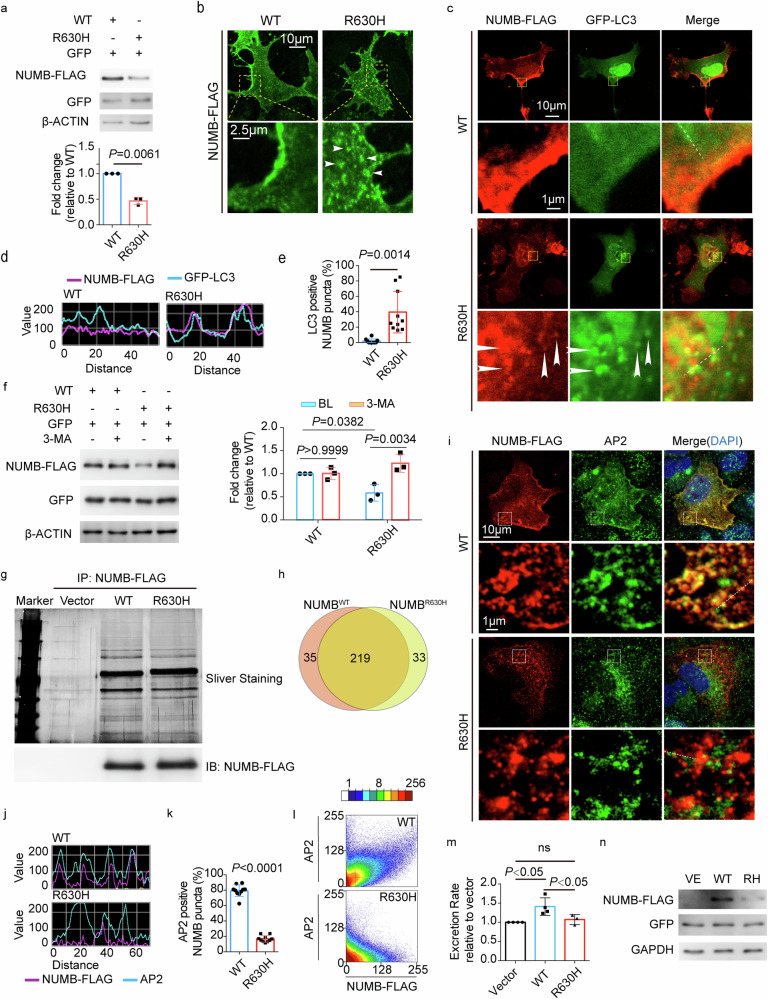
Abnormal intercellular distribution and excessive clearance of NUMB^R630H^ via autophagy. **a** Expression of NUMB^WT^ and NUMB^R630H^protein. WT NUMB-3FLAG or NUMB^R630H^-3FLAG plasmid was co-transfected with a vector expressing GFP into HEK293 cells, followed by detecting NUMB^WT^-FLAG and NUMB^R630H^-FLAG proteins. GFP and β-ACTIN were detected as the transfection and loading control, respectively. The detection was repeated three times. The statistic result is shown in the bottom panel (*n* = 3 per group). **b** The distribution of NUMB^WT^ and NUMB^R630H^ protein. HEK293 cells were transfected with NUMB^WT^-3FLAG or NUMB^R630H^-3FLAG plasmid, followed by immuno-fluorescence staining with FLAG tag antibody. The white arrow heads indicate the granule-like structures found in cells expressing NUMB^R630H^. **c**, **d** Immuno-fluorescence staining of NUMB^WT^ and NUMB^R630H^ and LC3. NUMB^WT^-3FLAG or NUMB^R630H^-3FLAG plasmid was co-transfected with GFP-LC3 plasmid into HEK293 cells, followed by immuno-fluorescence staining with FLAG tag antibody and GFP antibody. The white arrow heads indicate the granule-like structures of NUMB^R630H^ co-stained with LC3. Fluorescent signal intensity along the dashed lines are shown in **d**. **e** Comparison of the percentage of NUMB punctate or granule structure co-localized with LC3 between in cells expressing NUMB^WT^ and in cells expressing NUMB^R630H^ (*n* = 10 per group). **f** Detection of NUMB^WT^ and NUMB^R630H^ protein in cells treated with autophage inhibitor 3-MA. NUMB^WT^-3FLAG or NUMB^R630H^-3FLAG plasmid was co-transfected with vector expressing GFP. Treatment with 3-MA was started 36 h after transfection and last for 12 h. The expression of NUMB^WT^ and NUMB^R630H^ protein was detected using FLAG tag antibody and GFP was detected as a transfection control. The detection was repeated three times. The statistic results are shown in the right panel (*n* = 3 per group). **g**, **h** Interactome of NUMB^WT^ and NUMB^R630H^ protein. NUMB^WT^ and NUMB^R630H^-3FLAG plasmids were transfected into HEK293 cells. The co-immunoprecipitation was done using FLAG tag antibody. The precipitated proteins were separated by SDS-PAGE electrophoresis. The representative sliver staining image is shown in **g** upper panel. The immunobloting of immunoprecipitated NUMB^WT^ and NUMB^R630H^ protein are shown in **g** bottom panel. The Venn diagram of interacting proteins of NUMB^WT^ and NUMB^R630H^ protein is shown in **h. i** Immuno-fluorescence staining of NUMB^WT^, NUMB^R630H^ and AP2. HEK293 cells were transfected as mentioned above, followed by immuno-staining with FLAG tag antibody and anti-adaptin α antibody. **j** Fluorescent signal intensity along the dashed lines. **k** Statistical comparison of the percentage of NUMB punctate structure co-stained with AP2 between in cells expressing NUMB^WT^-FLAG and in cells expressing NUMB^R630H^-FLAG (*n* = 10 per group). **l** The scatterplot images of co-localization analysis between AP2 and NUMB^WT^ or NUMB^R630H^. **m** Statistical comparison of the excretion rate of uric acid among control MDCK cells (*n* = 4) and MDCK cells stably expressing NUMB^WT^ (*n* = 4) and NUMB^R630H^ (*n* = 3). **n** The expression of NUMB^WT^-FLAG and NUMB^R630H^-FLAG. MDCK cells were infected with control lentivirus (control, VE), lentivirus/ NUMB^WT^-FLAG or entivirus/NUMB^R630H^-FLAG, followed by selection with puromycin for 3 weeks. GFP and GAPDH were detected as the infection control and loading control, respectively. Statistics was calculated using two-tailed unpaired Student’s *t*-test (**a**, **e**, **k**), Two-way ANOVA with Tukey’s Multiple Comparison Test (**f**) or One-way ANOVA with Tukey’s Multiple Comparison Test (**m**). The result is presented as mean ± SD.

To study the functional impacts of NUMB^R630H^ on uric acid transport, we first expressed the NUMB^R630H^ mutant in a human kidney embryonic HEK293 cells and polarized MDCK cells. Detection of wild-type (WT) NUMB protein (NUMB^WT^) and NUMB^R630H^ mutant proteins by immunoblotting showed that after normalization with control GFP and β-actin protein levels, approximate 50% reduction of NUMB^R630H^ mutant protein was expressed relative to NUMB^WT^ protein (Fig. 4a). Immunocytochemical localization using the FLAG-tag demonstrated that the intracellular NUMB^R630H^ mutant protein was evenly distributed within the transfected cells and appeared as punctuated patterns (Fig. 4b). In some of the NUMB^R630H^ mutant-transfected cells, punctuated cluster signals were accumulated in the perinuclear region of the transfected cells (Fig. 4c). By contrast, NUMB^WT^ exhibited cell membrane and small-dotted staining patterns, which completely lacked the large granule-like structures seen in the NUMB^R630H^ mutant-transfected cells (Fig. 4b, c). Interestingly, the membrane distribution of NUMB^WT^ protein was accumulated on one-side of the plasma membrane, whereas opposite sides showed weak signals (Fig. 4b, c). These findings demonstrate that both the expression levels and cellular distribution of NUMB^R630H^ mutant protein are fundamentally different from NUMB^WT^ protein. This abnormality in the distribution of NUMB^R630H^ mutant protein was also found in the transfected MDCK cells (Supplementary Fig. S5a).

As protein aggregates are often cleared by the process of autophagy, we next investigated the relation between NUMB^R630H^ mutant protein and autophagy to explain the reduced protein levels in the transfected cells. Notably, the NUMB^R630H^ mutant protein was largely co-contained with autophagy-specific proteins, including the LC3-II mature autophagesome marker in HEK293 cells (Fig. 4c–e) as well as in MDCK cells (Supplementary Fig. S5b, d). These colocalization findings suggest that autophagy is potentially entailed in NUMB^R630H^ mutant protein degradation. Consistent with this notion, incubation of the NUMB-transfected cells with an autophagy inhibitor 3-methyladenine (3-MA) that inhibited the autophagesome maturation by preventing the conversion from LC3 into LC3-II restored the NUMB^R630H^ mutant protein levels (Fig. 4f). These findings provide compelling evidence that the NUMB^R630H^ mutant protein aggregates undergo autophagy-mediated protein degradation.

To gain further mechanistic insights into the functional impact of mutant NUMB^R630H^, we investigated the interactome between NUMB^R630H^ and NUMB^WT^ using co-immunoprecipitation and mass spectrum approaches. A total number of 219 interactive proteins were discovered to be commonly shared by NUMB^WT^ and NUMB^R630H^ (Fig. 4g, h and Supplementary Table S3). AP2 was one of them (Supplementary Table S3) and NUMB is known to bind to the clathrin-mediated endocytosis AP2 adaptors through the conserved DPF motif^41^. AP2 is a multimeric protein that internalizes the cargo membrane proteins through the clathrin-mediated endocytosis. Noticeably, colocalization signals of NUMB^R630H^ and AP2 were markedly decreased relatively to that between NUMB^WT^ and AP2 (Fig. 4i–l). NUMB^WT^ and AP2 showed almost completely colocalized overlapping signals (Fig. 4i–l). Surprisingly, the NUMB^R630H^ mutant protein aggregates lacked colocalization with AP2 (Fig. 4i–l). While AP2-positive signals were evenly distributed as small-dotted structures, the NUMB^R630H^ mutant positive signals appeared as large aggregative structures that were separated from the AP2 positive signals (Fig. 4i–l). On the basis of these findings, it appeared that although R630H variant in NUMB adjacent to the DPF motif did not interfere with the physical interactive domains between NUMB and AP2, NUMB^R630H^ protein molecules tended to form aggregates compartmentalized within autophagesomal machineries without AP2.

There were 35 proteins exclusively interacting with NUMB^WT^ and 33 other proteins interacting with NUMB^R630H^ (Fig. 4g, h and Supplementary Table S3). In searching for potential interactive proteins with NUMB^R630H^, we focused our efforts on the NUMB^R630H^ interactive 33 proteins, some of which were molecular chaperons in the cytosol and mitochondria (CCT3, CCT8, HSPB1, and HSPD1) and the mitochondrial outer membrane TOMM22 involving in transferring the aggregate-prone proteins to the mitochondrion^49^. Although lacking direct evidence, these findings support the notion that the mutant NUMB^R630H^ protein is involved in misfolding and degradation.

### Gain-of-function of NUMB^R630H^

We next performed gain-of-function experiments by stably overexpressing human WT and NUMB^R630H^ mutant proteins in MDCK cells. Expectedly, the NUMB-overexpressing cells showed high levels of the NUMB^WT^ and NUMB^R630H^ mutant proteins in their respective stable cell lines (Fig. 4n). The NUMB^WT^-overexpressing cells exhibited the significantly augmented excretion relative to the vehicle-transfected control cells (Fig. 4m). Conversely, the NUMB^R630H^ mutant-overexpressing cells lacked the capacity to increase uric acid excretion (Fig. 4m). These findings suggest that NUMB proteins are primarily involved in uric acid excretion.

### Knock-in of NUMB^R630H^ mutant increases urate levels in serum

**Fig. 5:**
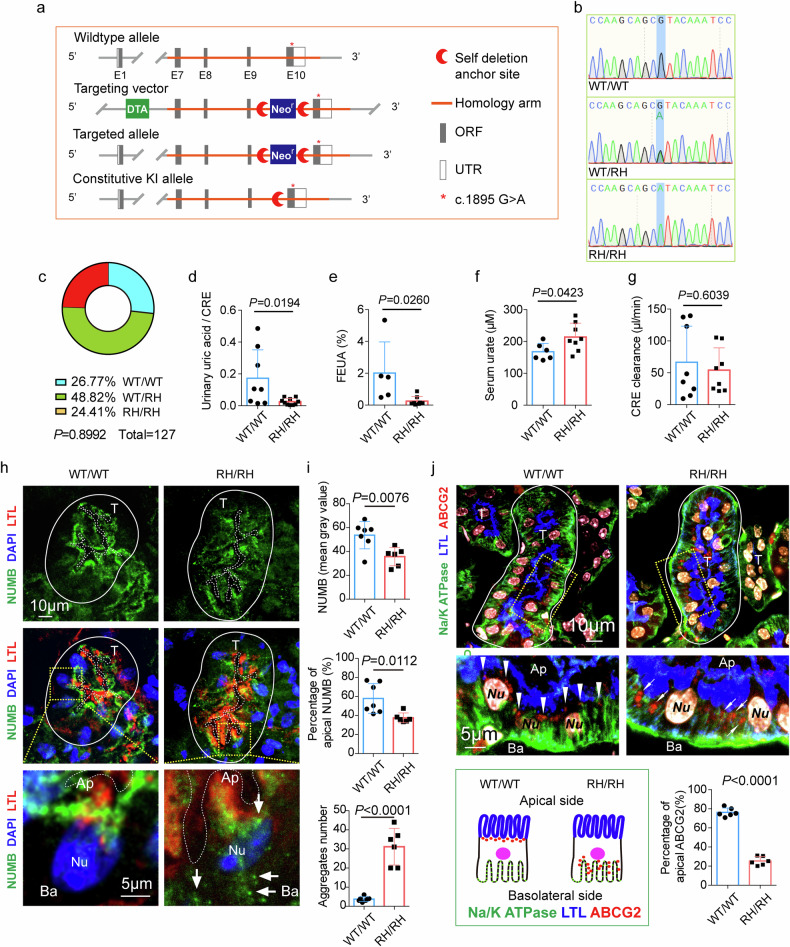
The defection in uric acid excretion and the abnormal distribution of NUMB and ABCG2 protein in renal tubular epithelia cells of NUMB^R632H^/NUMB^R632H^ homozygous mice. **a** Illustration of the establishment of orthologous NUMB^R632H^ mutation knock-in mice. **b** Representative electropherograms show the generation of mice heterozygous (WT/RH) or homozygous (RH/RH) for the introduced mutation (blue shadow). **c** Chi-square to compare observed and expected distributions of three genotype according to Mendel’s law. **d**–**g** Comparison of the ratio of urinary uric acid to urinary creatinine (**d**, WT/WT *n* = 8; RH/RH *n* = 10; *t* = 2.600, df = 16), the fractional excretion of uric acid (FEUA) (**e**, WT/WT *n* = 5; RH/RH *n* = 8; *t* = 2.570, df = 11), serum urate (**f**, WT/WT *n* = 6; RH/RH *n* = 8; *t* = 2.272, df = 12) and creatinine clearance (**g**, WT/WT *n* = 8; RH/RH *n* = 8; *t* = 0.5308, df = 14) between in WT/WT mice and in NUMB^R632H^/NUMB^R632H^ (RH/RH) mice. **h** Representative images of immunofluorescence staining of NUMB in kidney tissue of WT/WT mice and NUMB^R632H^ /NUMB^R632H^ mice. The solid lines indicate the edge of renal tubules, and the dashed lines mark the lumen of renal tubules. T: renal tubules; Ap: apical side of renal tubular epithelia cells (RTECs); Ba: basolateral side of RTECs. White arrows indicate sporadic distribution of granule-like structure of NUMB^R632H^ protein in cytoplasm of RTECs. **i** The statistic comparison of NUMB protein (top panel), apical NUMB (middle panel) and NUMB aggregate-like structure (bottom panel) each renal tubule between in WT/WT mice (*n* = 7) and in NUMB^R632H^/NUMB^R632H^ mice (*n* = 6). **j** Representative images of immunofluorescence staining of ABCG2 and basolateral marker Na/KATPase. The white arrow heads indicate the distribution of ABCG2 (red). In WT/WT mice, ABCG2 is distributed to the apical side just beneath the LTL signal. However, in NUMB^R632H^ /NUMB^R632H^ mice, ABCG2 located to the basolateral side with increased co-localization with Na/K ATPase. The illustration of ABCG2 distribution is shown in the bottom left, and the statistic result of the percentage of apical ABCG2 is shown in the bottom right (*n* = 6 per group, *t* = 19.34, df = 10). Statistics was calculated using two-tailed unpaired Student’s *t* test. The results are presented as mean ± SD.

To capitulate clinical hyperuricemia and gout, we attempted to generate an animal gout model by knocking in the NUMB^R630H^ mutant gene in mice. We took a homologous recombination approach to generate a mouse strain carrying the orthologous NUMB^R632H^ mutation in one allele, i.e., heterozygous mice (Fig. 5a). The WT/NUMB^R632H^ heterozygous mice were crossed each other to generate NUMB^R632H^/NUMB^R632H^ homozygous mice. The NUMB^R632H^ mutant gene in heterozygous and homozygous mice was frequently validated by sequencing analysis to ensure accuracy of animals used in our studies (Fig. 5b). Expectedly, the composition of WT/WT homozygosity, WT/NUMB^R632H^ heterozygosity, NUMB^R632H^/NUMB^R632H^ homozygosity was in concordance with Mendel’s Law (Fig. 5c).

Overall, WT/NUMB^R632H^ heterozygous and NUMB^R632H^/NUMB^R632H^ homozygous mice were alive without any fertility defects. These genetically manipulated mice showed indistinguishable bodyweights, blood pressures, fasting blood glucose levels and glucose tolerance test from those in WT/WT mice (Supplementary Fig. S6a–c, e). Interestingly, a slightly change in insulin tolerance test was detected in NUMB^R632H^/NUMB^R632H^ homozygous mice (Supplementary Fig. S6d). Together, these findings show that knock-in of the NUMB^R630H^ mutant gene in mice does not affect general physiology of mice.

Despite lack of overt phenotypes in WT/NUMB^R632H^ heterozygous and NUMB^R632H^/NUMB^R632H^ homozygous mice, uric acid levels in urine were significantly decreased in 48-week-old NUMB^R632H^/NUMB^R632H^ homozygous male mice (Fig. 5d). Accordingly, fractional excretion of uric acid (FEUA) was also reduced (Fig. 5e). In contrast, significant increases of serum urate levels were detected in NUMB^R632H^/NUMB^R632H^ homozygous male mice (Fig. 5f). However, excretion of creatinine by kidney remained unchanged (Fig. 5g), suggesting that decreased excretion of uric acid was not due to defective kidney functions. In female mice, we observed very similar phenotypes as those in male mice. Uric acid levels in urine were also significantly decreased in NUMB^R632H^/NUMB^R632H^ homozygous female mice (Supplementary Fig. S7c). Taken together, knock-in of the NUMB^R630H^ mutant gene in mice significantly impaired uric acid excretion by the kidney.

### Abnormal distribution of NUMB and ABCG2 in renal tubules

Immunofluorescence localization of NUMB protein in kidneys of both male and female mice uncovered apical assembly of NUMB protein in RTECs surrounding the tubular cavity (Fig. 5h and Supplementary Fig. S7a). Lotus tetragonolobus lectin (LTL) was used as an apical marker, which showed largely non-overlapping distribution with NUMB protein in NUMB^R632H^/NUMB^R632H^ homozygous mice (Fig. 5h, i middle panel; Supplementary Fig. S7a, b middle panel). These results show that NUMB protein in NUMBR632H/NUMBR632H homozygous mice was broadly distributed in RTECs and lacked apical assembly as seen in WT/WT mice. Moreover, the total cellular protein level of the NUMB^R632H^ mutant in the kidney tubules was remarkably lower than NUMB^WT^ (Fig. 5i top panel; Supplementary Fig. S7b top panel), which was consistent with the in vitro findings. In addition, NUMB^R632H^ mutant proteins formed large aggregate-like structures sparsely distributed in the cytoplasm of RTECs (Fig. 5h, i bottom panel; Supplementary Fig. S7a, b bottom panel) as seen in exogenously expressed human NUMB^R630H^ in HEK293 and MDCK cells, which was virtually absent in WT/WT mice (Fig. 5h, i bottom panel; Supplementary Fig. S7a, b bottom panel).

Similar to the abnormal distribution of NUMB protein, ABCG2 protein was also distributed in the lateral region of RTECs in NUMB^R632H^/NUMB^R632H^ homozygous mice (Fig. 5j). Quantification analysis showed that only 20% of positive signals of ABCG2 protein were located in the apical side and a majority of ABCG2 molecules were located in the basolateral sides. These findings reconcile with in vitro cell culture data in polarized MDCK cells.

### Chronic uric acid nephropathy in NUMB^R632H^/NUMB^R632H^ homozygous mice

**Fig. 6:**
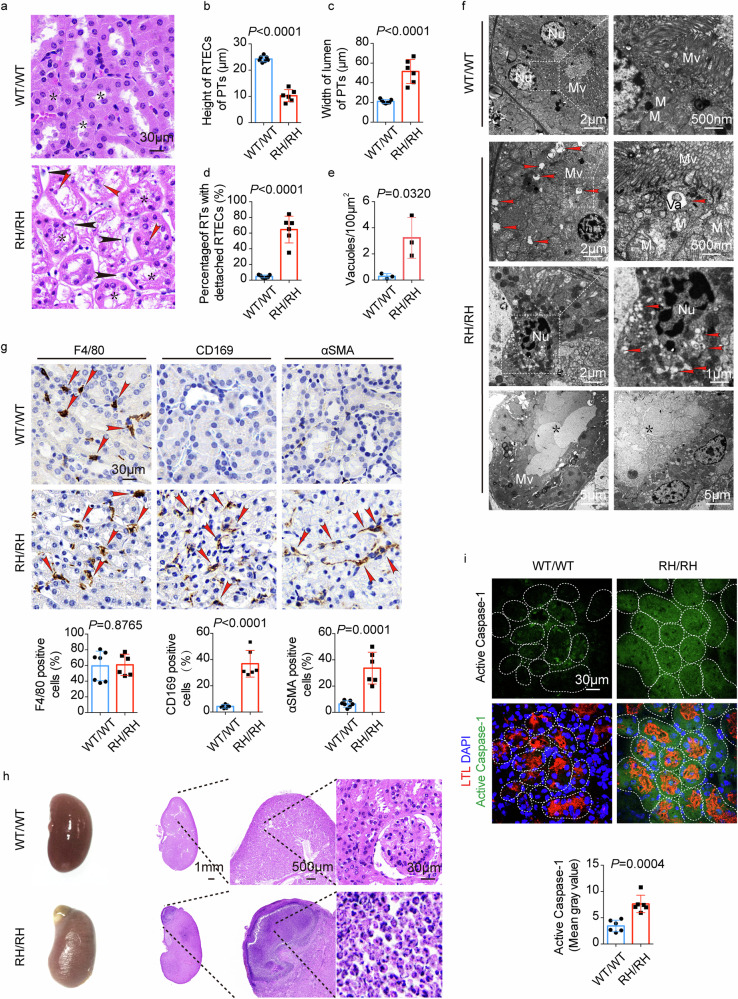
Chronic uric acid nephropathy in NUMB^R632H^ /NUMB^R632H^ homozygous mice. **a** H&E staining of the kidney tissue of WT/WT mice and NUMB^R632H^ /NUMB^R632H^ mice. The stars indicate the proximal tubules (PTs), the red arrow heads indicate the detached RTECs and the black arrow heads indicate the inflammatory cells infiltration. **b**–**d** Statistic comparison of the height of RTECs in PTs, the width of lumen of PTs and the percentage of renal tubules (RTs) with RTECs detached from basement membrane between in WT/WT mice (*n* = 7) and NUMB^R632H^ /NUMB^R632H^ mice (*n* = 6). **e**, **f** TEM images of renal tubules of WT/WT mice and NUMB^R632H^ /NUMB^R632H^ mice. Red arrow heads indicate vacuoles with diameter over 100 nm. Nu: nuclear; Mv: microvilli; M: mitochondria; Va: vacuole; star: non-cellular structures in the lumen. The statistic result of the amount of vacuoles with diameter over 100 nm per 100 μm^2^ is shown in **e** (*n* = 3, *t* = 3.228, df = 4). **g** Immunohistochemical staining of F4/80, CD169 and α-SMA. Red arrow heads indicate the positive cells. The statistic result of the percentage of F4/80^+^ (*n* = 6, *t* = 0.159, df = 11), CD169^+^ (*n* = 6, *t* = 8.428, df = 11) and α-SMA^+^ (*n* = 6, *t* = 5.870, df = 11) cells in total peritubular cells are shown in the bottom. **h** The representative gloss examination photos and H&E staining of renal tissue with severe inflammation in NUMB^R632H^ /NUMB^R632H^ mice. **i** Immunofluorescence staining of active Caspase-1. The tissues were also stained with LTL (red) and DAPI (blue). The statistic result of active Caspase-1 signal is show in the bottom panel (*n* = 6, *t* = 5.141, df = 10). Statistics was calculated using two-tailed unpaired Student’s *t* test (**b**–**g**, **i**). The results are presented as mean ± SD.

Since hyperuricemia and gout primarily cause damage in the renal tubules, we performed histological analysis of kidneys in WT/WT and NUMB^R632H^/NUMB^R632H^ homozygous mice. Intriguingly, a majority of number of NUMB^R632H^/NUMB^R632H^ homozygous mice showed disorganization, architectural, and structural changes of renal tubules (Fig. 6a). Typically, the average height of RTECs in the proximal tubules was markedly reduced (Fig. 6b) and the width of the lumen was enlarged (Fig. 6c) in 80-week-old NUMB^R632H^/NUMB^R632H^ homozygous mice relative to those in WT/WT mice at the same age. Damaged RTECs detached from basement membrane were frequently observed in those NUMB^R632H^/NUMB^R632H^ homozygous mice (Fig. 6d). In WT/WT kidneys, each of renal tubules was well-organized with the lumen in the central tubule surround by epithelial cells (Fig. 6a). In contrast, the lumen of each tubule in NUMB^R632H^/NUMB^R632H^ kidney was indistinct and filled with non-cellular structures (Fig. 6a). Transmission electron microscopic (TEM) analysis validated the existence of non-cellular structures within the lumen of renal tubules in NUMB^R632H^/NUMB^R632H^ kidney (Fig. 6f). Additionally, NUMB^R632H^/NUMB^R632H^ tubular epithelial cells contain a large number of vacuolar structures with diameter over 100 nm, which were almost completely lacking in WT/WT tubular epithelial cells (Fig. 6e, f). It appeared that most of the vacuolar structures enclose contents and accumulated beneath the apical microvilli of tubular epithelial cells (Fig. 6f). Increased infiltration of inflammatory monocytes/macrophages, especially CD169^+^ macrophages, and fibroblasts were also found in the tubular areas of NUMB^R632H^/NUMB^R632H^ kidney (Fig. 6g). In some NUMB^R632H^/NUMB^R632H^ kidneys, inflammation was visible at the gloss examination (Fig. 6h). Indeed, histological examination corroborated severe renal inflammation in NUMB^R632H^/NUMB^R632H^ kidney (Fig. 6h). The inflammatory cells exhibited Ly6G positivity, demonstrating the infiltration and accumulation of neutrophils (Supplementary Fig. S8a). Noticeably, accumulation of F4/80^+^ monocytes/macrophages was found around the inflammatory areas (Supplementary Fig. S8b). A recent study shows that hyperuricemia triggers activation of Caspase-1 in RTECs^50^. Consistently, high level of the activated Caspase-1 was detected in NUMB^R632H^/NUMB^R632H^ RTECs (Fig. 6i). These findings provide structural basis of nephropathy in NUMB^R632H^/NUMB^R632H^ mice. Also, accumulation of intracellular vacuoles beneath the apical microvilli in NUMB^R632H^/NUMB^R632H^ tubules suggests defective exchanges of molecule cargos between RTECs and the renal tubule lumen.

## Discussion

Increased production of uric acid, decreased excretion of uric acid, and mixed type of both are the common causes of hyperuricemia and gout. Kidney and liver play prominent roles in regulating serum urate levels^26^. Defective excretion often entails kidney disease, drugs, and competition of uric acid excretion with other molecules^7^. Genome-wide association analysis revealed genetic variants in genes encoding uric acid transporters related with the serum urate level and occurrence of gout. At least 10% of all gout cases in persons of European ancestry are attributable to a loss of function variant of ABCG2^Q141K^, which influence ABCG2 expression in a tissue-specific manner and is responsible for extra-renal uric acid under-excretion^51,52^.

In this study, we identified a relatively large gout family that exhibited typically heterozygosity in the affected family members. Whole-exome sequencing analysis of 5 gout and hyperuricemia individuals identified a missense variant of *NUMB* commonly present in all gout-affected family members tested. Owing to the heterozygosity of the genetic aberration, the decreased levels of the WT NUMB is sufficient to cause hyperuricemia and gout. This means that optimal expression levels of the NUMB protein are necessary to sustain the hemostatic function of uric acid metabolism. Alternatively, the NUMB^R630H^ mutant protein possesses a novel function to interfere with the WT NUMB for uric acid metabolism. NUMB known as a clathrin adaptor protein plays a crucial role in determining the cell fate and specification during development^30,41,43^. A recent study has shown that NUMB safeguards the apical sorting of EAAT3 transporter in polarized cells by binding to the YVNGGF motif located in the C-terminus of EAAT3 protein^44^. Genetic silencing NUMB or replacement of the YVNGGF motif with other amino acids resulted in anomalous localization of EAAT3. Similar to that study, we identified a conserved NUMB-binding YxNxxF motif in the N-terminus of ABCG2, which is a key membrane transporter for uric acid excretion. Using co-immunoprecipitation and colocalization approaches, we provide compelling evidence of physical interaction between NUMB and ABCG2.

The key function of WT NUMB protein is to safeguard apical sorting of ABCG2 and reduced NUMB would dislocate ABCG2 trafficking to the apical membrane, where excretion of uric acid occurs. The NUMB^R630H^ mutant protein molecules form aggregates and undergo degradation by the autophagesome machinery. Thus, the total level of intracellular NUMB protein is decreased and the NUMB^R630H^ mutant protein is considered as a nonfunctional variant. It is unclear why NUMB^R630H^ mutant protein undergoes degradation through an autophagy-mediated mechanism. We reasonably speculate that the NUMB^R630H^ mutant protein molecules are misfolded and become recognized by the autophagesomal machinery. In this regard, NUMB^R630H^ mutant protein no longer catches ABCG2 for apical sorting. This interesting aspect warrants further investigation. The dysfunctional consequence of ABCG2 transporter caused by reduced NUMB proteins reflects the fact that an optimal ratio between these two proteins is required for excretion of uric acid by ABCG2. Along this view, any factors, including genetic or attained factors that alter the optimal ratio between NUMB and ABCG2 would potentially cause hyperuricemia and gout.

A formidable experiment of using genetic knock-in of orthologous mutant NUMB^R632H^ in mice recapitulated the phenotype of hyperuricemia seen in human patients. These findings provide compelling evidence that the mutant NUMB^R630H^ protein plays a causative role in developing hyperuricemia. Consistent with in vitro polarized RTECs culture studies, colocalization of ABCG2 and various cell markers demonstrated decreased apical distribution of ABCG2, leading to markedly reduced urine excretion and serum accumulation of urate. Similar to human hyperuricemia and gout patients, NUMB^R632H^ homozygous mice developed a chronic uric acid nephropathy. Although the formation of urate crystals in joints and kidneys is not obvious in these mouse models, inflammation is overwhelmed in the affected kidney tissues, especially the infiltration of CD169 positive inflammatory cells. This important point needs clinical validation.

This genetic knock-in mouse model not only provides a powerful tool to study the mechanism underlying uric acid excretion, but potentially serves as a platform for drug development. In theory, if an agent differentially upregulates WT NUMB or downregulates the functionally inactive mutant NUMB, this agent would potentially be developed as a drug for treating NUMB-deficient patients.

Since gout is one of the most common diseases, understanding molecular mechanisms underlying gout development is inevitably pivotal to define therapeutic targets. Our present work uncovers an imperceptible mechanism underlying the causative link between NUMB and hyperuricemia/gout. This function of the intracellular protein NUMB in uric acid excretion is unprecedentedly unknown and unobvious. Together, our findings provide novel mechanistic insights into the complex interaction of NUMB with a membrane transporter in polarized cells in determining gout development. On the basis of these findings, it is highly probable that NUMB may also participate in apical trafficking of other member transporters as seen in EAAT3. If the NUMB-mediated apical sorting is a general mechanism, NUMB defects would causatively trigger development of multiple disorders and alterations of drug responses. Therefore, normalization of NUMB function provides an exciting and attractive approach for treating hyperuricemia/gout and possible other human diseases.

## Materials and methods

### Materials, plasmids, and cells

HEK293 and MDCK cells were obtained from Type Culture Collection of the Chinese Academy of Sciences and maintained with DMEM medium supplemented with 10% FBS and MEM medium supplemented with 10% FBS, respectively. NUMB2-eGFP-M107K was a gift from Rolf Bjerkvig^53^ (Addgene plasmid # 37803). Plasmids NUMB2-R630H-eGFP-M107K, NUMB2-3FLAG-M107K and NUMB2-R630H-3FLAG-M107K were generated using the template NUMB2-eGFP-M107K. Plasmid ABCG2-3FLAG (Ubi-ABCG2-3FLAG-CBh-gcGFP-IRES-puromycin), Lentivirus/NUMB-WT-3FLAG (Ubi-NUMB-WT-3FLAG-CBh-gcGFP-IRES-puromycin), Lentivirus/NUMB-R630H-3FLAG (Ubi-NUMB-R630H-3FLAG-CBh-gcGFP-IRES-puromycin) and the corresponding control lentivirus were obtained from Genechem (Shanghai, China). Plasmid ABCG2-eGFP-pcDNA3.1 + C-eGFP was obtained from Genscript (Nanjing, China).

### Patients

The family with inherited gout and the 300 Chinese patients with sporadic gout were recruited from the Affiliated Hospital of Qingdao University (Qingdao, China). All patients were clinically diagnosed with primary gout according to the diagnostic criteria established by the American Society of Rheumatology in 1997, and the secondary gout was excluded. The 300 sporadic gout patients recruited included 297 males and 3 females. Age at examination ranged from 16 to 83 years with a median of 47 years. A hundred unrelated healthy individuals, without hyperuricaemia (serum urate (SUA) levels > 7.0 mg/dL) or history of gout were recruited from the Affiliated Hospital of Qingdao University (Qingdao, China). The healthy individuals recruited including 57 males and 43 females with age at examination ranged from 40 to 81 years with a median of 52 years. Human kidney specimen were obtained from a patient with kidney stone taking partial nephrectomy. This study was approved by Ethical Committee of the Affiliated Hospital of Qingdao University (QYFY WZLL 28875). All protocols were in accordance with the Declaration of Helsinki, and written informed consent was obtained from all participants.

### Whole-exome sequencing

Whole-exome sequencing (WGS) was performed in 5 patients and 3 healthy individuals from the gout family. Briefly, 1 μg genomic DNA was randomly fragmented by Covaris. The fragmented was selected by Agencourt AMPure XP-Medium kit to an average size of 150-250 bp. The selected fragments were subjected to end-repairing, 3’ adenylating, adapters-ligation, PCR Amplifying and the products were recovered by the AxyPrep Mag PCR clean up Kit. Certain amount of PCR product was taken for hybridization with BGI Hybridization and Wash kit and recovered with AxyPrep Mag PCR clean up Kit. The double-stranded PCR products were heat denatured and circularized by the splint oligo sequence. The single-strand circle DNA (ssCir DNA) was formatted as the final library. The qualified libraries were amplified to make DNA naoball which had more than 300 copies of one molecular. The DNA naoball were loaded into the pattered naoarray and pair-end 100 bases reads were generated in the way of sequenced by combinatorial Probe-Achor Sythesis on BGISEQ-500 platform (BGI-Shenzhen, china).

### Polymerase chain reaction and Sanger sequencing of NUMB

The genomic region spanning SNP rs374597310 (*NUMB*) and SNP rs2231142 (A*BCG2*) was amplified from genomic DNA by Polymerase chain reaction (PCR) followed by Sanger sequencing. The PCR primers to amplify SNP flanking region are as following: SNP rs374597310 (forward (5’- GCTACCACCAGTCCCTTCTT -3’); reverse (5’- GTTTTGCTCCTTTGACCGCT -3’)); SNP rs2231142 (forward (5’- ACTGCAGGTTCATCATTAGCT -3’); reverse (5’- GACCCTGTTAATCCGTTCGT -3’)). Sanger sequencing was performed by BGI Tech (Shenzhen, China).

### CRISPR/Cas9-mediated gene knockout

Single guide RNA (sgRNA) targeting Human *NUMB* exon 4 (5’- GATGAAGAAGGCGTTCGCAC -3’), exon 7 (5’- GTGACAGATCCAGCGACGAG -3’), or Dog *NUMB* (5’- GATGAAGAAGGGGTTCGTAC -3’) were cloned into LentiCRISPRv2 vector. To generate *NUMB*-knockout HEK293 cells, LentiCRISPRv2 carrying sgRNA against human *NUMB* were transfected into HEK293 cells and selected with puromycin (Cat. No. P8230, Solarbio China) for 14 days. Cells transfected with empty LentiCRISPRv2 vector was used as the negative control. To generate *NUMB*-knockout MDCK cells, MDCK cells was infected with lentivirus/ LentiCRISPRv2-Dog *NUMB* sgRNA, followed by selection with puromycin for 14 days. Cells infected with control lentivirus was used as the negative control.

### In vitro uric acid transport assay

The in vitro uric acid transport assay was conducted according to the previous report^54^ with minor modifications. Briefly, MDCK cells were grown in the transwell inserts with 0.4 μm pores (Cat. No. 3460, Corning Incorporated). After reaching 100% confluence, the cells were maintained for another 5 days to establish a polarized monolayer. Uric acid excretion and reabsorption measurements were initiated by adding transport buffer (HBSS with CaCl_2_ and MgSO_4_) containing ^15^N-uric acid (1,3-[^15^N]2 uric acid, Cat. No. 62948-75-8, Cambridge Isotope Laboratories) to the donor side and transport buffer without ^15^N-uric acid to the receiver side. To determine the integrity of MDCK monolayers, Lucifer yellow (Cat. No. L0144, Sigma-Aldrich) was added together with ^15^N-uric acid. The cells with neither ^15^N-uric acid nor Lucifer yellow added to the donor side were set as the blank. The inhibitor was added to only the apical side 30 min before the initiation of the measurement. An aliquot of transport buffer was obtained from the donor side at 5 min for measurement of initial concentration, and from the receiver side at 240 min. Transport studies were performed at 37 °C. The concentration of ^15^N-uric acid was determined by UPLC-mass spectrum (UPLC I-CLASS, Waters; AB 4500 q-traq, AB Sciex). The excretion rate and the reabsorption rate were calculated as follows:

The excretion or the reabsorption rate (%) = (concentration of ^15^N-uric acid in transport buffer of receiver side × volume of transport buffer of receiver side) / (initial concentration of ^15^N-uric acid of in transport buffer of donor side × volume of transport buffer of donor side) × 100%.

### Immunoblot

Cells were lysed in pre-cold RIPA lysis buffer (25 mM Tris-HCl,150 mM NaCl,1% NP40,1% Sodium deoxycholate,0.1% SDS, pH 7.6) containing proteinase and phosphatase inhibitor cocktails (Cat. No. HYK0010, Med Chem Express, China; Cat. No. HYK0021, Med Chem Express, China; 1:100). Equal amount of protein samples and a standard molecular weight marker (Cat. No. WJ102, EpiZyme, China) were loaded on a 10% SDS-PAGE gel, followed by electrophoresis and transferring onto a polyvinylidene difluoride membrane (Cat. No. 10600023, GE Healthcare Life Science). The membranes were subsequently blocked with 5% skimmed milk for 1 h and probed overnight at 4 °C with a rabbit anti-NUMB antibody (Cat. No. 2756, Cell Signaling; 1:1000), a mouse anti-NUMB antibody (Cat. No. sc-136554, Santa cruz; 1:200), a mouse anti-GFP antibody (Cat. No. 632381, Clontech; 1:2000), a rabbit anti-GFP antibody (Cat. No. G1544, Sigma; 1:1000), a mouse anti-FLAG tag antibody (Cat. No. F1804, Sigma; 1:1000), a rabbit anti-FLAG tag antibody antibody (Cat. No. F7425, Sigma; 1:1000), and a mouse anti-β-actin antibody (Cat. No. A5441, Sigma; 1:10000) in 5% skimmed milk. After rigorous washing with PBS containing 0.1% Triton X-100, membranes were incubated at room temperature for 1 h with a goat anti-mouse IgG horseradish peroxidase (HRP)-conjugated antibody (Cat. No. 115-035-062, Jackson ImmunoResearch; 1:10000) and a goat anti-rabbit IgG HRP-conjugated antibody (Cat. No. 111-035-144, Jackson ImmunoResearch; 1:10000). Target proteins were visualized using a Chemiluminescent HRP substrate reagent (Cat. No. WBKLS0100, Millipore) with a Molecular Imager (ImageQuant LAS500, GE).

### Co-immunoprecipitation

Cells were lysated with pre-cold lysis buffer (1% TritonX-100, 10 mM HEPES, pH 7.4, 142.5 mM KCl, 5 mM MgCl_2_, 1 mM EDTA, 10% Glycerol) containing proteinase and phosphatase inhibitor cocktails and centrifuged at 10000× *g*, 4 °C for 10 min. The protein concentration in the supernatant was determined using BCA protein assay kit (Cat. No. 23225, Thermo Scientifc). Equal amount of protein (1–1.5 mg) was incubated with FLAG tag antibody (Cat. No. F1804, Sigma; 1:500) and Protein G Sepharose beads (Cat. No. 17-0618-01, GE Healthcare) overnight at 4 °C. The beads was washed four times by spinning down at 500× *g* for 2 min and resuspending with pre-cold washing solution (1% TritonX-100, 10 mM HEPES, pH 7.4, 142.5 mM KCl, 5 mM MgCl_2_, 1 mM EDTA). The proteins were denatured by boiling in Laemmli buffer (63 mM Tris-HCl, 2% SDS, 10% glycerol, 5% β-mercaptoethanol, 0.0025% Bromophenol blue, pH 6.8) and separated with SDS-PAGE electrophoresis.

### Liquid chromatography-tandem mass spectrometry

The co-immunoprecipitation samples were denatured in 8 M urea in 30 mM NH_4_HCO_3_, reduced with 10 mM Dithiothreitol (DTT) and alkylated by 15 mM iodoacetamide (IAA). Subsequently, the proteins were digested with trypsin at 37 °C overnight and desalted using C18 column. The peptides were separated by Easy-nLC 1200 ultra performance liquid chromatography (UPLC) and then analyzed using Q Exactive high resolution mass spectrometer. Proteome Discover 2.5 software were used for protein and peptide identification. The liquid chromatography-tandem mass spectrometry (LC-MS/MS) analysis was performed by Luming Bio (Shanghai, China).

### Immunofluorescence

Cells grown on coverslips were fixed with 4% paraformaldehyde for 15 min, permeablized in PBS containing 0.1% Saponin for 10 min and blocked with 5% BSA. The cells were subsequently incubated for 1 h at room temperature with the primary antibodies included a rabbit anti-NUMB antibody (Cat. No. 2756, Cell Signaling; 1:200), a mouse anti-NUMB antibody (Cat. No. sc-136554, Santa cruz; 1:200), a mouse anti-FLAG tag antibody (Cat. No. F1804, Sigma; 1:200), a rabbit anti-EEA1 antibody (Cat. No. 3288, Cell Signaling; 1:200), a rabbit anti-RAB11 antibody (Cat. No. 5589 T, Cell Signaling; 1:100), a rabbit anti-RAB7 antibody (Cat. No. 9367, Cell Signaling; 1:100), a goat anti-ZO-1 antibody (Cat. No. PA5-19090, Invitrogen, 1:200), a mouse anti-Na/K ATPase antibody (Cat. No. 05-369, Millipore; 1:200), a rabbit anti-Na/K ATPase antibody (Cat. No. A11683, ABclonal; 1:200), a rabbit anti-β CATENIN antibody (Cat. No. 9582, Cell Signaling; 1:200), a mouse anti-GFP antibody (Cat. No. 632381, Clontech; 1:200) and a mouse anti-adaptin α antibody (Cat. No. 610501, BD Biosciences; 1:200). All the secondary antibodies were obtained from Jackson ImmunoResearch which included: a donkey anti-mouse IgG Alexa 647 antibody (Cat. No. 715-606-150 1:100), a donkey anti-rabbit IgG cy3 antibody (Cat. No. 715-166-152, 1:400) and a donkey anti-mouse IgG Alexa488 antibody (Cat. No. 715-546-150, 1:400), a donkey anti-goat IgG DyLight 405 antibody (Cat. No. 705-475-147 1:100). Nuclei were stained with DAPI (Cat. No. D9542, Sigma). The images were captured using a confocal fluorescence microscope (SPE, Leica).

### Detection of cell surface ABCG2

Cells were washed with ice-cold PBS/CM (PBS containing 1 mM MgCl_2_ and 1.3 mM CaCl_2_) for three times and then incubated in fresh-made solution of 0.25 mg/mL Sulfo-NHS-SS-Biotin (Cat. No. PG82077, Pierce) in PBS/CM for 30 min at 4 °C. The reaction was quenched by incubating cells in 50 mM NH_4_Cl in PBS/CM at 4 °C for 10 min. The cells were then lysated with lysis buffer (0.2% SDS, 1% Triton X-100, 0.5% Deoxycholic acid, 50 mM Tris-HCl, pH 8.0, 150 mM NaCl, protease inhibitors freshly added) and brief sonication. The biotinated proteins were purified with NeutrAvidin Agarose (Cat. No. 29201, Pierce) for 1 h at room temperature. The biotin label was then removed by incubating the purified biotinated protein with 50 mM DTT in sample buffer (2% SDS, 62.5 mM Tris-HCl, pH 6.8, 10% glycerol) for 1 h at room temperature. Surface ABCG2 was then detected by western blot.

### Animals

WT/NUMB^R632H^ heterozygous mice in the C57BL/6 background were generated by Cyagen Biotechnology Co., Ltd. (Suzhou, China) through homologous recombination. The orthologous R632H (CGT to CAT) mutation was introduced into exon 10 of mouse *Numb* gene located on mouse chromosome 12 (NCBI Reference Sequence: NM_001136075.2). Adult males and female mice were caged at 22 ± 3 °C under 12-h–12-h light–dark cycles with food (standard chew diet) and water provided ad libitum. All the WT/WT and NUMB^R632H^/ NUMB^R632H^ mice tested were generated by crossing the WT/ NUMB^R632H^ heterozygous mice to each other. The primers for genotyping are provided in Supplementary Table S4.

Renal excretion of uric acid was evaluated using metabolic cages (Suzhou Feng’s Laboratory Animal Equipment, China). The mice were singly housed in metabolic cages supplied with water only. The water consumption was recorded and 24-h urine was collected. The blood samples were collected from submandibular vein. The concentration of urinary uric acid and creatinine, serum urate and creatinine were determined using uric acid (Cat. No. C011-2-1, Nanjing Jiancheng, China) and creatinine detection kit (Cat. No. C012-2-1, Nanjing Jiancheng, China). FEUA was calculated by dividing the product (urinary uric acid × serum creatinine) by the product (serum urate × urinary creatinine) and multiplying the result by 100%.

All of the mouse studies were approved by the Animal Ethical Committee of Qingdao University, Qingdao, China. The experimenters were not blinded to the assignment of the groups and the evaluation of the results. No samples, animals, or data were excluded.

### Histology, immunohistochemistry, and frozen section immunofluorescence

Paraffin-embedded tissues were cut in 5 μm thickness, deparaffinized in Xylene, and sequentially rehydrated in 99%, 95%, 70%, and 50% ethanol. Tissue slides were counterstained with H&E before dehydration with 95% and 99% ethanol, and were mounted with balsam neutral. For Immunohistochemical staining, paraffin-embedded tissue section was deparaffinized, rehydrated, heated at 97 °C in epitope retrieval solution (Cat. No. DM828, DM818, dako) for 20 min and incubated with 3% H_2_O_2_. The tissue sections were stained with a rabbit anti-F4/80 antibody (Cat. No. GB11027, Servicebio, China; 1:500); a rabbit anti-CD163 antibody (Cat. No. GB113751, Servicebio, China, 1:500), a rabbit anti-CD169 antibody (Cat. No. GTX131703, Gene Tex, 1:200); a rabbit anti-αSMA antibody (Cat. No. 19245, Cell Signaling; 1:200). The secondary antibody was HRP-conjugated goat anti-rabbit antibody (Cat. No. ab6721, Abcam, 1:250).

For immunofluorescence staining, frozen tissue sections with 8 μm thickness were fixed with 4% paraformaldehyde for 15 min, permeablized in PBS containing 0.1% TritonX-100, blocked with a blocking solution containing 5% donkey serum (Cat. No. abs935, Absin, China) and 2.5% BSA. For immunostaining with a mouse IgG antibody, the tissue section was then blocked with an unconjungated goat anti-mouse IgG (Cat. No. ab6668, Abcam; 0.1 mg/mL) before incubated with the primary antibodies. The primary antibodies included: a rabbit anti-NUMB antibody (Cat. No. 2756, Cell Signaling; 1:200), a rabbit anti-ABCG2 antibody (Cat. No. 42078, Cell Signaling; 1:200), a mouse anti-Na/K ATPase antibody (Cat. No. 05-369, Millipore; 1:200), and a rabbit anti-cleaved CASPASE-1 antibody (Cat. No. 89332, Cell Signaling; 1:100). The tissue section was subsequently stained with the proximal tubular marker lotus tetragonolobus lectin (biotinated, Cat. No.1325, Vector Labs, 1:100), washed with PBS three times and probed with streptavidin-cy5 (Cat. No., Vector Labs, 1:100) and the secondary antibodies mentioned above.

### Statistics

Statistical analyses were performed with Prism 6 software (GraphPad). Student’s *t*-test (unpaired, two-tailed) was used to determine the significance of the difference between 2 groups. One-way ANOVA with Tukey’s Multiple Comparison Test were used to assess the difference among 3 groups. Two-way ANOVA with Tukey’s Multiple Comparison Test or Sidak Multiple Comparison Test were employed to estimate the changes according to the levels of two categorical variables. *χ*^2^ test was used to test for compliance with Mendel’s law.

## Supplementary information

Supplementary information, Figures and Tables

Raw data

Supplementary video S1

Supplementary video S2

## Data Availability

All data described in this work are present either in the main text or in the Supplementary information. The sequence data obtained by WES have been deposited in the China National GeneBank DataBase (https://db.cngb.org/) under Project ID CNP0006119.
