# Supplementary material for: NUMB dysfunction defines a novel mechanism underlying hyperuricemia and gout
Source: Cell Discov. 2024 Oct 22;10:106. doi: 10.1038/s41421-024-00708-6 (PMC11494200; doi:10.1038/s41421-024-00708-6)
Supplement: Supplementary file 1 — Supplementary information, Figures and Tables [file 41421_2024_708_MOESM1_ESM.pdf]

Supplementary Information

Supplementary Table S1 Demographic information of the gout patients and a subset of healthy individuals

Patient NO.	Gender	Age	Gout	HUA	Age of onset	Height (cm)	Weight (kg)	BMI (kg/m^2)	Urea nitrogen (mmol/L)	Creatinine (μmol/L)	Glucose (mmol/L)	Triglyceride (mmol/L)	Cholesterol (mmol/L)
Ⅱ-5	M	89	Yes	Yes	60	166	85	30.8	6.17	106.00	5.02	1.56	4.97
Ⅲ-3	F	72	Yes	Yes	50	160	80	31.3	5.04	56.00	13.33	2.56	6.23
Ⅲ-17	F	65	Yes	Yes	53	158	70	28.0	5.21	47.00	5.43	1.71	5.91
Ⅲ-19	M	63	Yes	Yes	43	176	72	23.2	5.07	93.00	4.89	2.06	4.65
Ⅲ-21	F	62	Yes	Yes	58	160	66	25.8	3.77	58.00	5.64	1.90	5.42
Ⅲ-23	F	59	Yes	Yes	47	155	53	22.1	6.26	72.00	5.88	0.89	6.51
Ⅲ-25	M	53	Yes	Yes	40	184	85	24.2	4.64	87.00	6.34	1.41	5.92
Ⅳ-2	M	45	Yes	Yes	38	175	78	25.5	4.90	68.00	10.67	3.60	4.52
Ⅳ-34	M	32	Yes	Yes	31	180	85	26.2	4.52	74.00	4.82	1.87	5.12
Ⅳ-30	F	38	No	Yes	30	163	58	21.8	4.95	50.00	4.60	0.58	4.64
Ⅲ-9	M	66	No	No		160	88	29.0	4.11	72.00	5.49	1.41	6.10
Ⅲ-13	F	62	No	No		150	63	27.8	6.80	93.40	4.20	2.41	6.78
Ⅲ-15	F	59	No	No		150	45	20.0	6.20	86.40	4.00	2.56	5.37
Ⅳ-4	M	42	No	No		173	74	24.8	4.94	68.00	11.61	4.60	4.52
Ⅳ-14	M	43	No	No		162	60	22.9	6.90	96.20	3.80	0.95	5.74
Ⅳ-18	M	39	No	No		165	70	25.7	6.80	87.10	3.70	1.14	3.61
Ⅳ-27	M	42	No	No		165	85	31.2	7.30	98.30	3.80	2.21	5.07
Ⅳ-32	F	34	No	No		166	65	23.5	5.82	66.00	11.05	2.12	5.29

Supplementary Table S2 Clinical characteristics of gout patients

Patient NO.	Urinary calculi (w/wo)	Tophi (w/wo)	Highest serum urate (μmol/L)	Urate at onset (μmol/L)	Annu onset frequency	Joints affected	Hypertension (w/wo)	Diabetes (w/wo)	Hyperlipidemia (w/wo)	Coronary heart disease (w/wo)
Ⅱ-5	w	w	502	464	10-12	4	w	wo	wo	w
Ⅲ-3	wo	wo	518	403	2-3	2	wo	w	w	w
Ⅲ-17	wo	wo	487	360	2-3	2	w	wo	wo	wo
Ⅲ-19	wo	w	591	457	5-6	3	w	w	wo	wo
Ⅲ-21	wo	wo	468	392	2-3	1	w	wo	wo	wo
Ⅲ-23	wo	wo	509	302	1-2	1	wo	wo	wo	wo
Ⅲ-25	w	wo	519	355	3-4	2	wo	wo	wo	wo
Ⅳ-2	wo	wo	462	462	1-2	1	wo	wo	w	wo
Ⅳ-34	wo	wo	453	453	2	1	wo	wo	wo	wo

Supplementary Table S3 Proteins interact with NUMB^{WT} AND NUMB^{R630H}

Proteins interact with both NUMB^{WT} and NUMB^{R630H}						Proteins interact with NUMB^{WT}	Proteins interact with NUMB^{R630H}
ACOT9	EPS15	MRPS22	RPL13	RPS12	SLC25A3	AKAP8	AHSG
ANAPC7	EPS15L1	MRPS23	RPL13A	RPS13	SMAP	BYSL	ANKRD28
AP1B1	FAM120A	MRPS24	RPL14	RPS14	SNRNP70	CAD	ANXA2
AP2A1	FAU	MRPS25	RPL15	RPS15	SNRPB	CALM3	ATP8B1
AP2A2	GEMIN4	MRPS26	RPL17	RPS15A	SNRPD2	CCT5	CCT3
AP2B1	GNL3	MRPS27	RPL18	RPS16	SNRPD3	CSNK2B	CCT8
AP2M1	GPRC5A	MRPS28	RPL18A	RPS17	SNRPE	DDX41	CDC16
AP2S1	H1-3	MRPS31	RPL19	RPS18	SNRPG	EIF3H	CDC27
ATAD3B	H2BC18	MRPS33	RPL22	RPS19	SNX9	IGLV1-51	DCAF7
ATP5MF	H3-7	MRPS34	RPL23	RPS2	SRSF1	KPNA2	DDB1
AURKAIP1	HACD3	MRPS35	RPL23A	RPS20	SRSF3	KRT6B	EEF1A1
BAG2	HADHA	MRPS5	RPL24	RPS21	SRSF6	MRPL49	ELP1
BANF1	HNRNPA0	MRPS6	RPL26	RPS23	SRSF9	MRPS18C	EPPK1
CCT2	HNRNPL	MRPS7	RPL27	RPS24	SSB	PDCD4	GAPDH
CCT4	HNRNPM	MRPS9	RPL27A	RPS25	SSBP1	PGAM5	GIMAP4
CDC23	HNRNPU	NAP1L1	RPL28	RPS26	SUPT16H	PKD2	GTF3C1
CFL1	HNRNPUL2	NCL	RPL3	RPS27L	SURF6	PNO1	HADHB
CHCHD1	ILF2	NPM1	RPL30	RPS28	TCOF1	PRMT5	HSD17B12
CLTC	IPO7	NUMB	RPL31	RPS29	TRA2B	RPL21	HSPB1
CSNK2A1	KANK2	NUMBL	RPL32	RPS3	TSR1	RPL29	HSPD1
DAP3	KHDRBS1	PARP1	RPL34	RPS3A	TUFM	SKP1	LUC7L2
DDX17	KPNA1	PPP2R1A	RPL35	RPS4X	U2AF1	SMARCA5	MT-CO2
DDX5	KRT8	PPP2R2A	RPL35A	RPS5	U2AF2	SPAG9	NCOR1
DHX15	LTV1	PPP6R3	RPL36	RPS6	UBR5	SQSTM1	PHKA2

DYNLL1	MACROH2A1	PRDX4	RPL36AL	RPS7	USP9X	SRP72	PHKG2
EEF2	MATR3	PRKDC	RPL4	RPS8	WDR26	SRSF7	PPP6C
EIF3A	MOV10	PTCD3	RPL5	RPS9	XRN2	SUGP2	SEC61A1
EIF3B	MRPS10	PYCR3	RPL6	RPSA	YBX1	SYNCRIP	SLC25A1
EIF3CL	MRPS11	RACK1	RPL7	RSF1	YWHAB	TBC1D32	TOMM22
EIF3D	MRPS12	RALY	RPL7A	RUVBL1	YWHAE	TBL1XR1	TRIM28
EIF3E	MRPS14	RBM39	RPL8	RUVBL2	YWHAQ	UACA	TUBAL3
EIF3F	MRPS15	RBMX	RPLP0	SAFB	ZC3H4	USP7	YWHAG
EIF3G	MRPS16	RCN2	RPLP1	SERBP1	ZC3HAV1	WDR82	ZNF572
EIF3I	MRPS17	RNGTT	RPLP2	SET	ZFR	YBX3	
EIF3L	MRPS18B	RPL10A	RPS10	SLC25A10	ZNF326	YPEL5	
EIF3M	MRPS2	RPL11	RPS11	SLC25A11	ZNRD2	YTHDC2	
EIF4A1	MRPS21	RPL12					

Supplementary Table S4. Genotyping Primers

Type		Sequence (5'-3')	Annealing temperature (℃)
5'arm-sdneo cassette PCR	Forward	GCCTGTAACTCGAGGGACCTA	62
	Reverse	CCCTAGCAGAGAGACTGGCTATAA	
5'arm-sdneo cassette PCR	Forward	AGCCTTGTTTTGTAGCCTGTAAC	62
	Reverse	GACTCTGCTTTAGTGGGATGAGA	
sequencing PCR	Forward	CAATGGTTCTGCAGCTTTCAATG	62
	Reverse	GATCTGGAAAGCAAACAAAGTCCC	

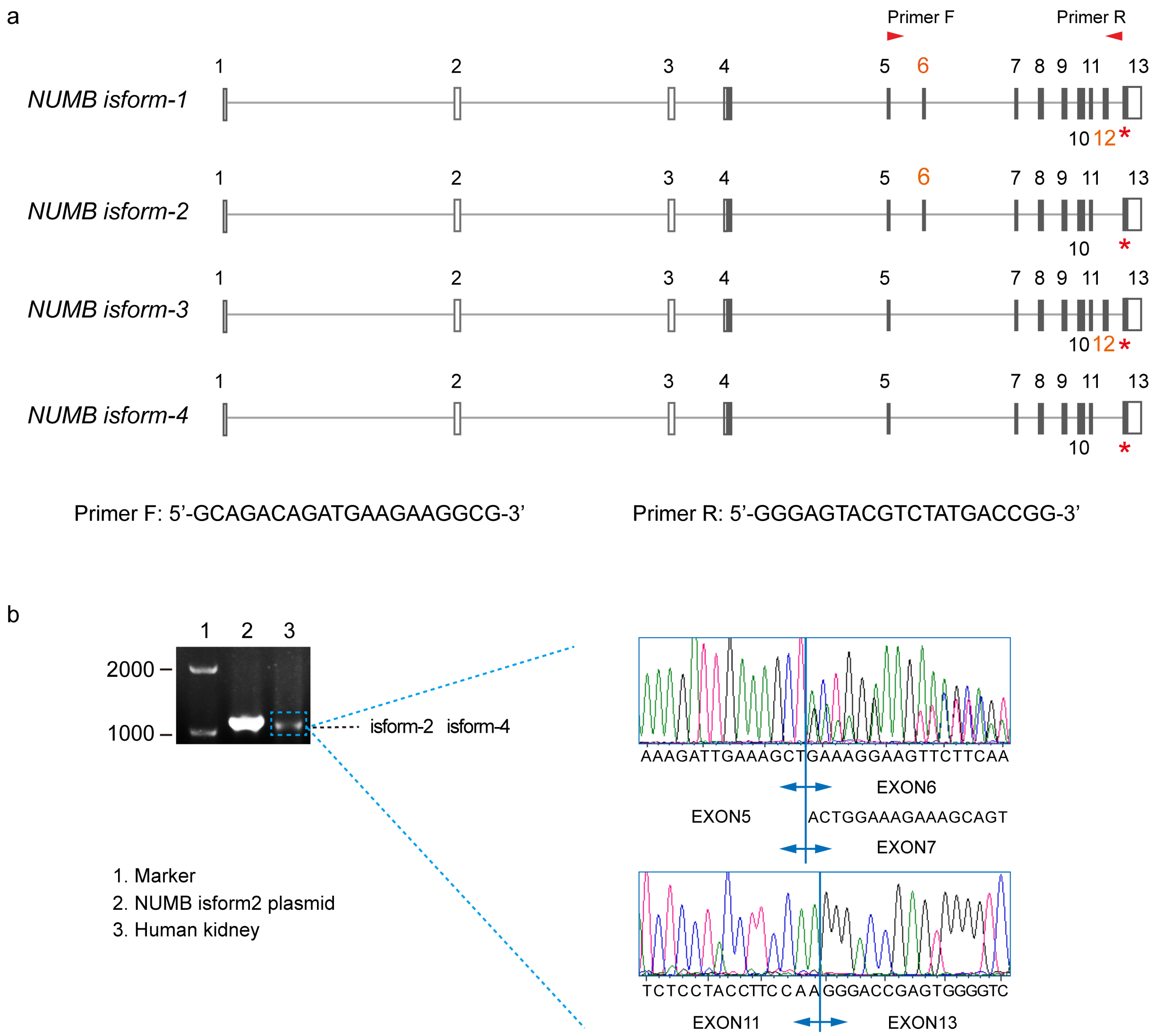

Supplementary Fig. S1 NUMB isforms in human kidney. **a,** The diagram of NUMB isforms and the primers used to amplify the gDNA region covering the known splicing site. The red triangles indicate the location of the primers. *, *NUMB* variant (rs375597310). **b,** The agarose gel electrophoresis of the PCR product using NUMB isform-2 plasmid as template (2) and RT-PCR products of human kidney tissue (3). The band marked with dashed line rectangle was cut and sequenced. The results are shown in the right panel.

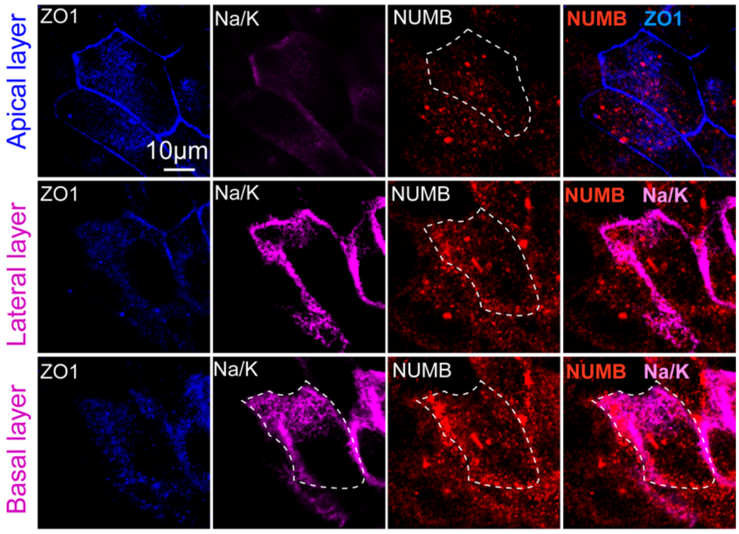

Supplementary Fig. S2 The distribution of NUMB in polarized MDCK cells. Immunofluorescence staining of NUMB in polarized MDCK cells. ZO-1 (blue), Na/K ATPase (magenta) were stained. NUMB (red) was immuno-stained with mouse anti-NUMB antibody (Santa Cruz).

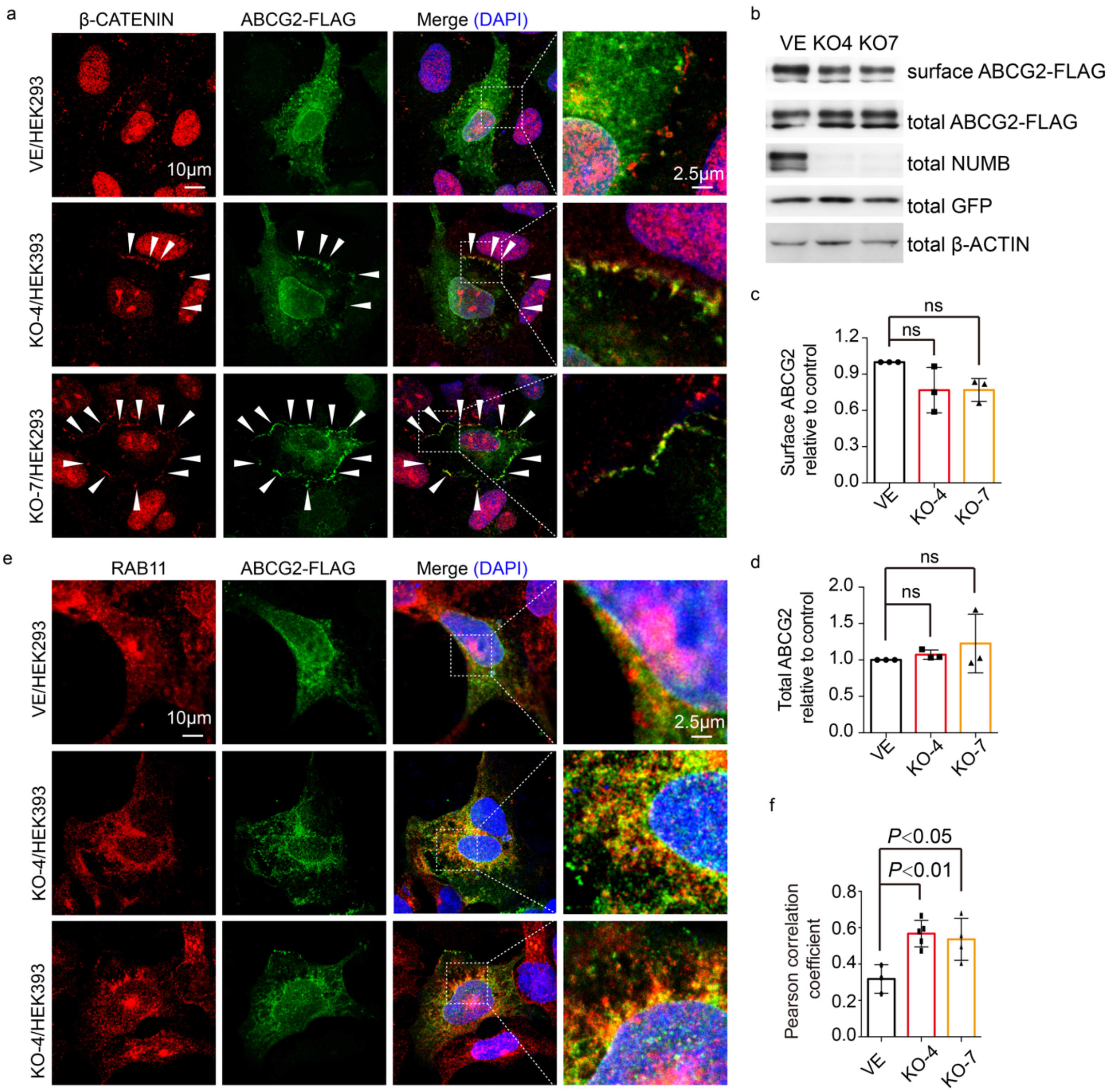

Supplementary Fig. S3 Distribution of ABCG2 in *NUMB* knockout HEK293 cells. **a,** Distribution of ABCG2 in *NUMB* knockout or control HEK293 cells. ABCG2-FLAG plasmid was transfected into *NUMB* knockout (KO) or control (VE) HEK293 cells, followed by immunofluorescence staining with FLAG tag antibody and β-CATENIN antibody. The white arrow heads indicate the accumulation of ABCG2 protein at the cell-cell contact region close to the adhesive junction. KO-4, KO-7 indicate knockout cells generated by sgRNA targeting exon 4 and exon 7, respectively. **b-d,** Detection of surface ABCG2 protein and ABCG2 in whole cell lysate (total

ABCG2) in *NUMB* knockout or control HEK293 cells. The statistic result is shown in **c-d** (n=3 per group). **e-f,** The co-localization of ABCG2 with RAB11 in *NUMB* knockout or control HEK293 cells. The statistic result is shown in **f** (VE n=3; KO-4 n=5; KO-7 n=4)**.** Statistcs was calculated with One-way ANOVA with Tukey's Multiple Comparison Test. The results are presented as mean ± SD.

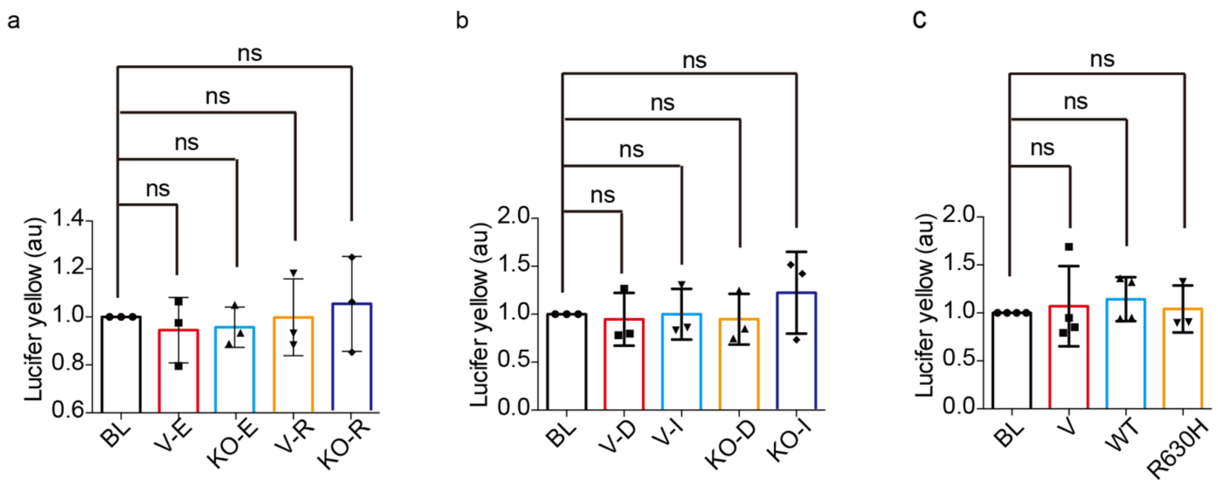

Supplementary Fig. S4 Determination of the integrity of MDCK monolayers in trans-cellular uric acid transport assay. **a,** Statistic result of the fluorescence of Lucifer yellow in the receiver side of control cells (V-E) and NUMB KO cells (KO-E) in the excretion assay, and of control cells (V-R) and NUMB KO cells (KO-R) in the reabsorption assay. BL: blank control. (n=3 per group). **b,** Statistic analysis on the fluorescence of Lucifer yellow in the receiver side of control cells treated with ABCG2 inhibitor (V-I) or with the solvent DMSO (V-D), and *NUMB* KO cells treated with ABCG2 inhibitor (KO-I) or with the solvent DMSO (KO-D) in the excretion assay (n=3 per group). **c,** Statistic comparison of the fluorescence of Lucifer yellow between in the receiver side of blank control (n=4) and of control cells (V, n=4), the $NUMB^{WT}$ stably expressing MDCK cells (WT, n=4) or the $NUMB^{R630H}$ stably expressing MDCK cells (KO, n=3) in the excretion assay. Statistcs was calculated with One-way ANOVA with Tukey's Multiple Comparison Test. The results are presented as mean ± SD.

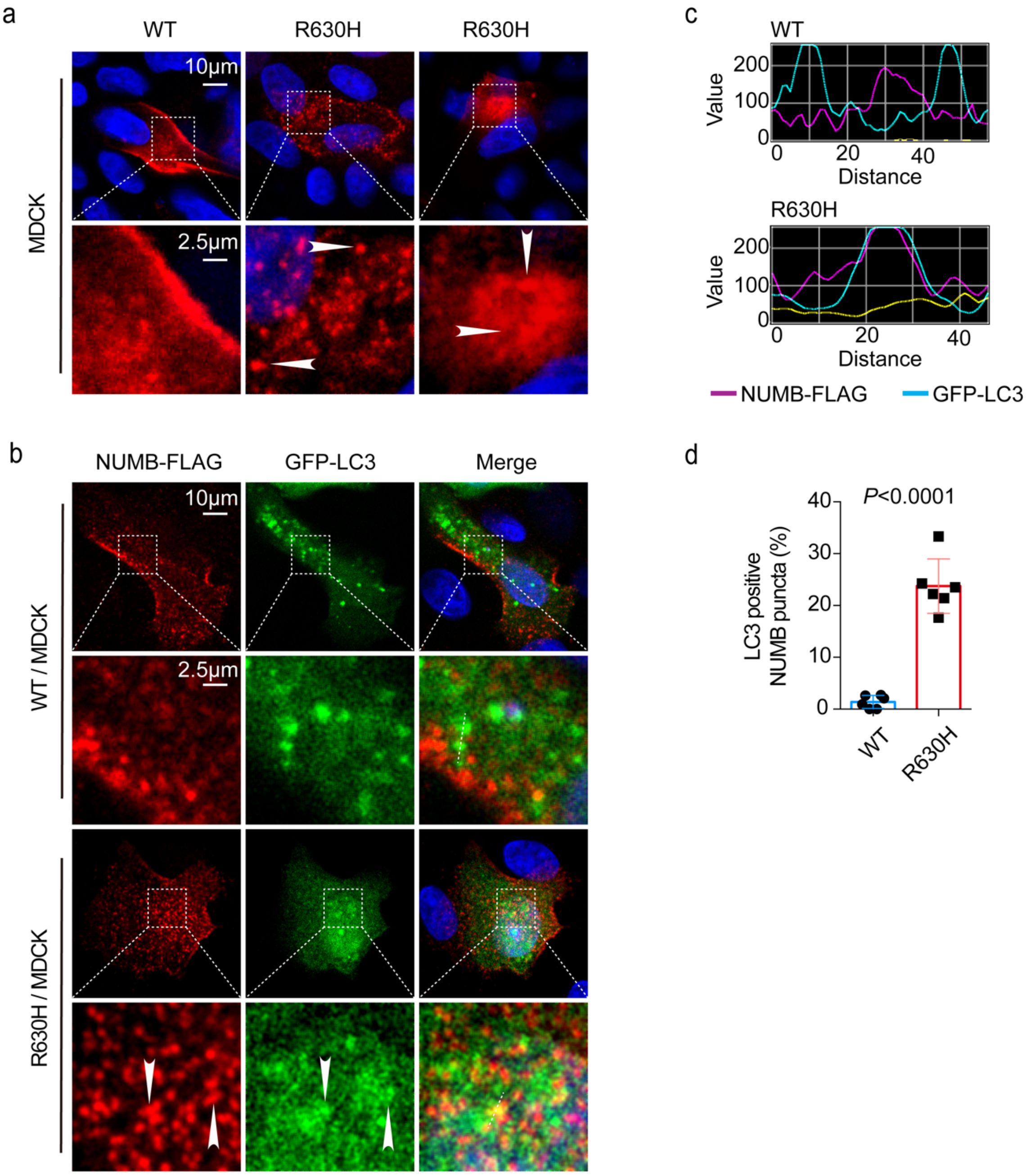

Supplementary Fig. S5 Abnormal distribution of NUMB^{R630H} mutant protein and its co-localization with LC3 in MDCK cells. **a,** The distribution of NUMB^{WT} and NUMB^{R630H} protein in MDCK cells. MDCK cells were transfected with NUMB^{WT}-3FLAG or NUMB^{R630H} -3FLAG plasmid, followed by immuno-fluorescence staining with FLAG tag antibody. The white arrow heads indicate the granule-like structure found in cells expressing NUMB^{R630H}. **b-d,** Immuno-fluorescence staining of

NUMB^{WT} and NUMB^{R630H} and LC3. NUMB^{WT}-3FLAG or NUMB^{R630H} -3FLAG plasmid was co-transfected with GFP-LC3 plasmid into MDCK cells, followed by immuno-fluorescence staining with FLAG tag antibody and GFP antibody. The white arrow heads indicate the granule-like structures of NUMB^{R630H} co-stained with LC3. Fluorescent signal intensity along the dashed lines are shown in **c**. **d,** Comparison of the percentage of NUMB punctate or granule structure co-localized with LC3 between in cells expressing NUMB^{WT} and in cells expressing NUMB^{R630H} (n=6 per group). Statistics was calculated using two-tailed unpaired Student's *t* test. The results are presented as mean ± SD.

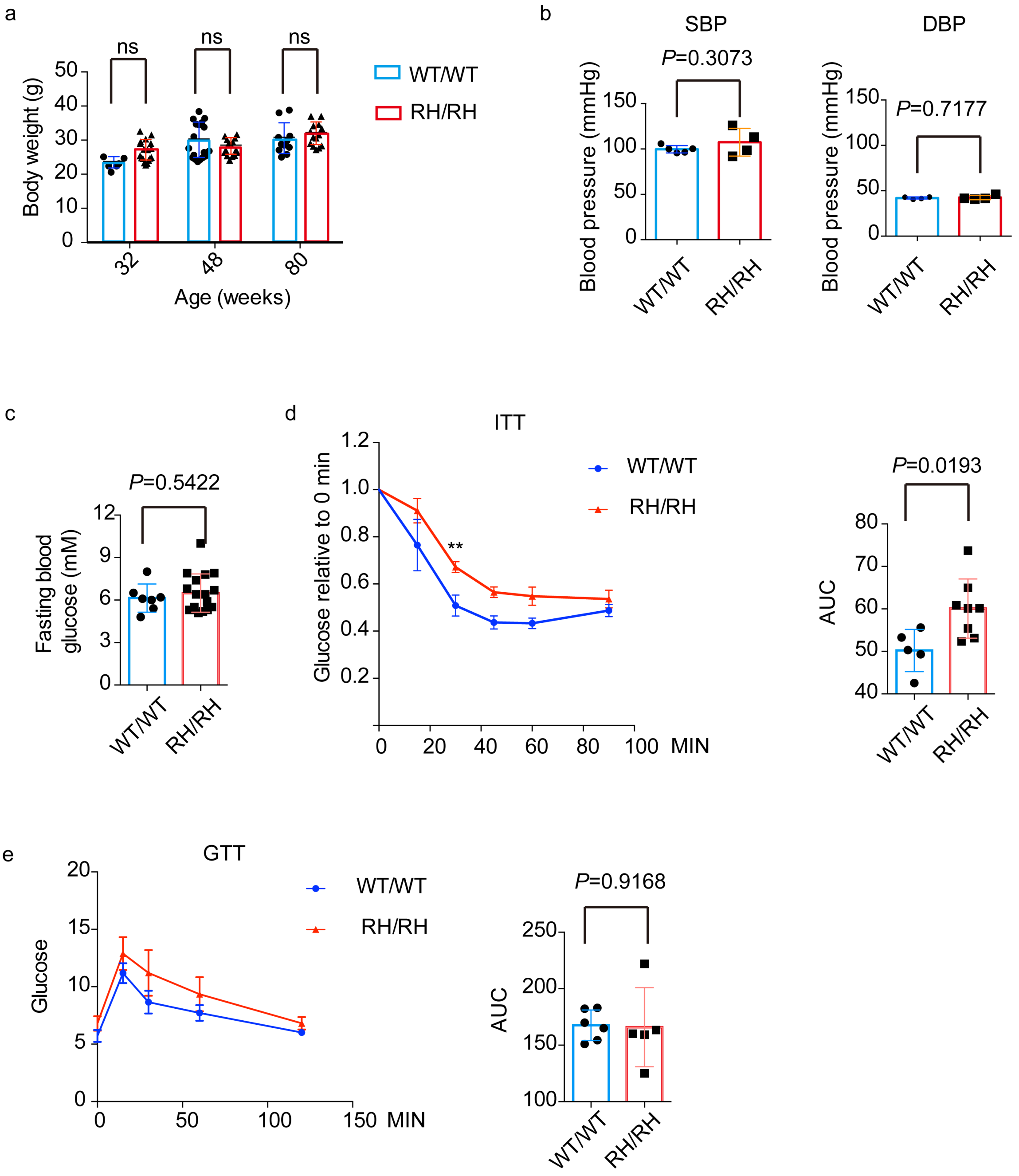

Supplementary Fig. S6 Knockin of the Numb^{R630H} mutant gene in mice does not affect general physiology of mice. a, Comparison of body weight of WT/WT mice and NUMB^{R632H} /NUMB^{R632H} mice at the age of 32 (WT/WT n=6, RH/RH n=18), 48 (WT/WT n=18, RH/RH n=15) and 80 weeks (WT/WT n=11, RH/RH n=14)**. b,** The blood pressure of 80-weeks WT/WT mice and NUMB^{R632H} /NUMB^{R632H} mice. SBP (WT/WT n=5, RH/RH n=14; t =1.101, df = 7) and DBP (n=4 per group, t =0.3790, df

= 6) represent systolic and diastolic blood pressure, respectively. **c,** The fasting blood glucose of 80-weeks WT/WT mice (n=7) and NUMB^{R632H} /NUMB^{R632H} mice (n=17; *t* =0.6192, df = 22). **d-e,** The insulin tolerance test (ITT) and glucose tolerance test (GTT) of 80-weeks WT/WT mice and NUMB^{R632H} /NUMB^{R632H} mice. The statistic results of area under curve of ITT (WT/WT n=5, RH/RH n=8; *t* =0.2738, df = 11) and GTT (WT/WT n=6, RH/RH n=5; *t* =0.1074, df = 9) are shown in the right panel. Statistics was calculated using two-tailed unpaired Student's *t* test (**b-e**) or Two-way ANOVA with Sidak's Multiple Comparison Test (**a**). The results are presented as mean ± SD.

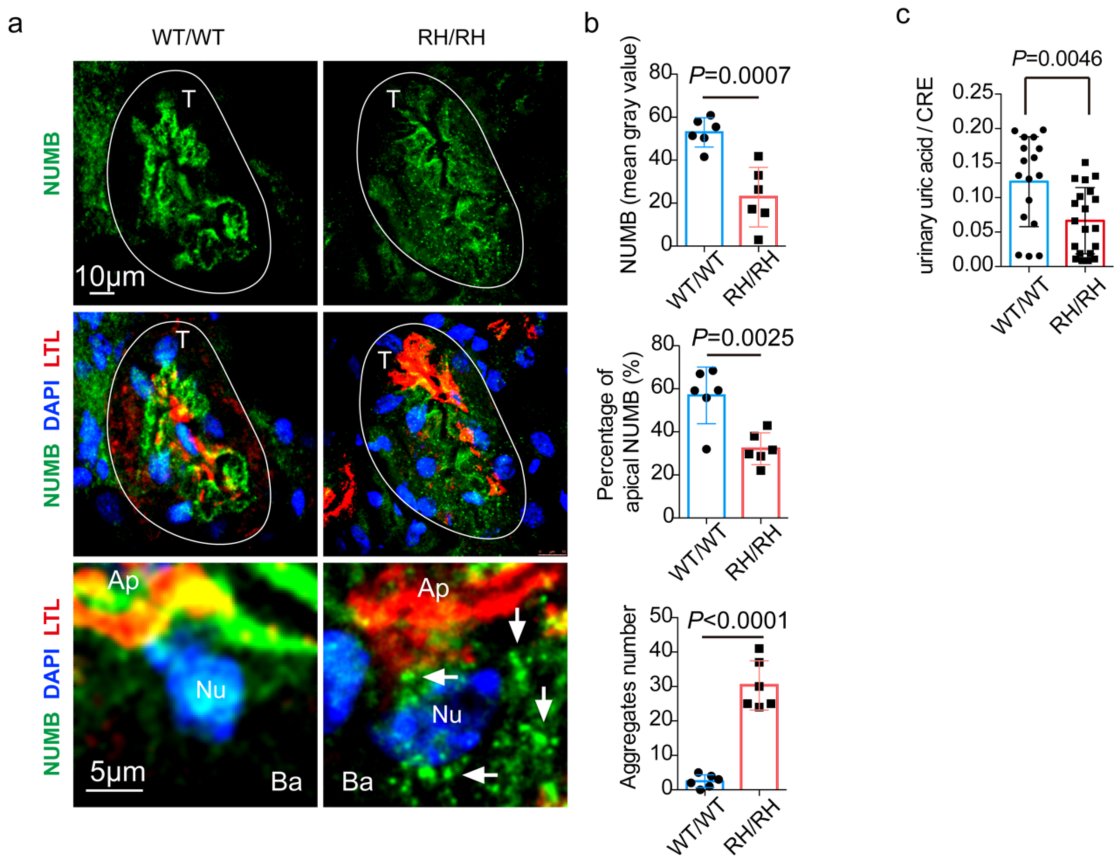

Supplementary Fig. S7 The abnormal distribution of NUMB^{R632H} in renal tubular epithelia cells and the defection in uric acid excretion of female NUMB^{R632H} /NUMB^{R632H} homozygous mice. a, Representative images of immunofluorescence staining of NUMB in kidney tissue of female WT/WT mice and NUMB^{R632H} /NUMB^{R632H} mice. The solid lines indicate the edge of renal tubules. T: renal tubules; Ap: apical side of RTECs; Ba: basolateral side of RTECs. White arrows indicate sporadic distribution of granule-like structure of NUMB^{R632H} protein in cytoplasm of RTECs. **b,** Statistic comparison of NUMB protein (top pannel), apical NUMB (middle panel) and NUMB aggregate-like structure (bottom panel) each renal tubule between in female WT/WT mice (n=6) and in female NUMB^{R632H} /NUMB^{R632H} mice (n=6). **c,** Comparison of the ratio of urinary uric acid to urinary creatinine between in female WT/WT mice and in female NUMB^{R632H}/NUMB^{R632H} (RH/RH) mice

(WT/WT n=17; RH/RH n=20). Statistics was calculated using two-tailed unpaired Student's *t* test. The results are presented as mean ± SD.

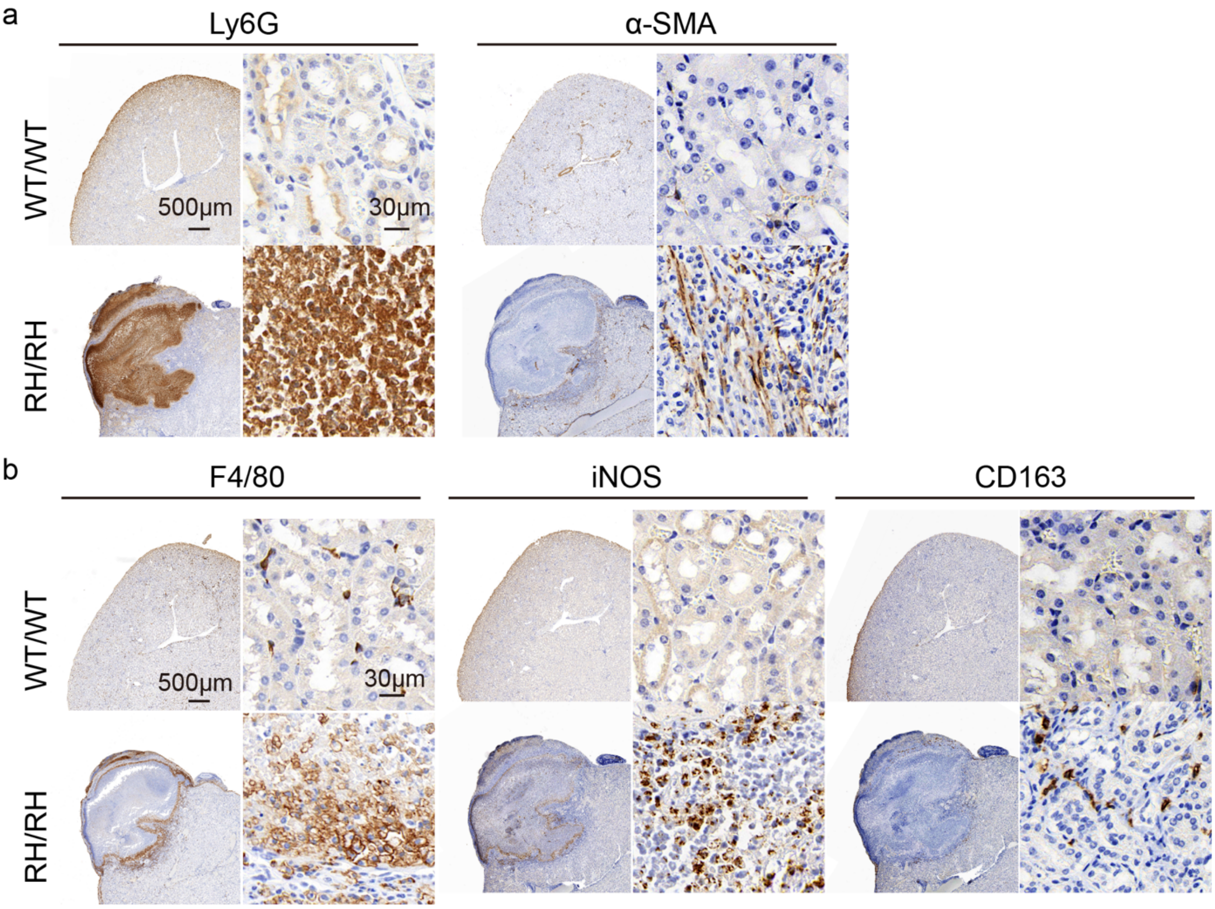

Supplementary Fig. S8 Immunohistochemical staining of renal tissue with severe inflammation in NUMB^{R632H} /NUMB^{R632H} mice. a, Immunohistochemical staining of neutrophils marker Ly6G and myofibroblast marker α-SMA. **b,** Immunohistochemical staining of pan macrophages marker F4/80, M1 type macrophages marker iNOS and M2 type macrophages marker CD163.

Supplementary Video S1 **3D projection showing the distribution of ABCG2 in control polarized MDCK cells.**

Supplementary Video S2 **3D projection showing the distribution of ABCG2 in *NUMB*-knockout polarized MDCK cells.**
