# Supplementary material for: NUMB dysfunction defines a novel mechanism underlying hyperuricemia and gout
Source: Cell Discov. 2024 Oct 22;10:106. doi: 10.1038/s41421-024-00708-6 (PMC11494200; doi:10.1038/s41421-024-00708-6)

Raw data for Figure 2b-c

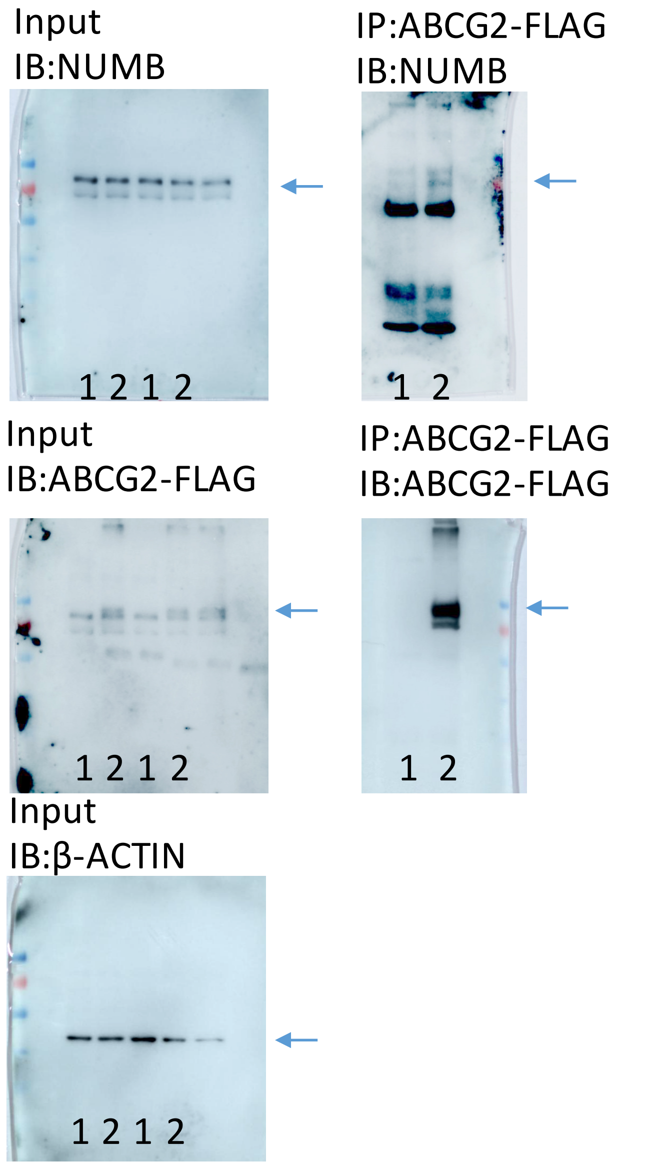

IP with FLAG antibody in HEK293 cells transfected with empty vector (negative control 1) and those transfected with ABCG2-FLAG plasmid (2)

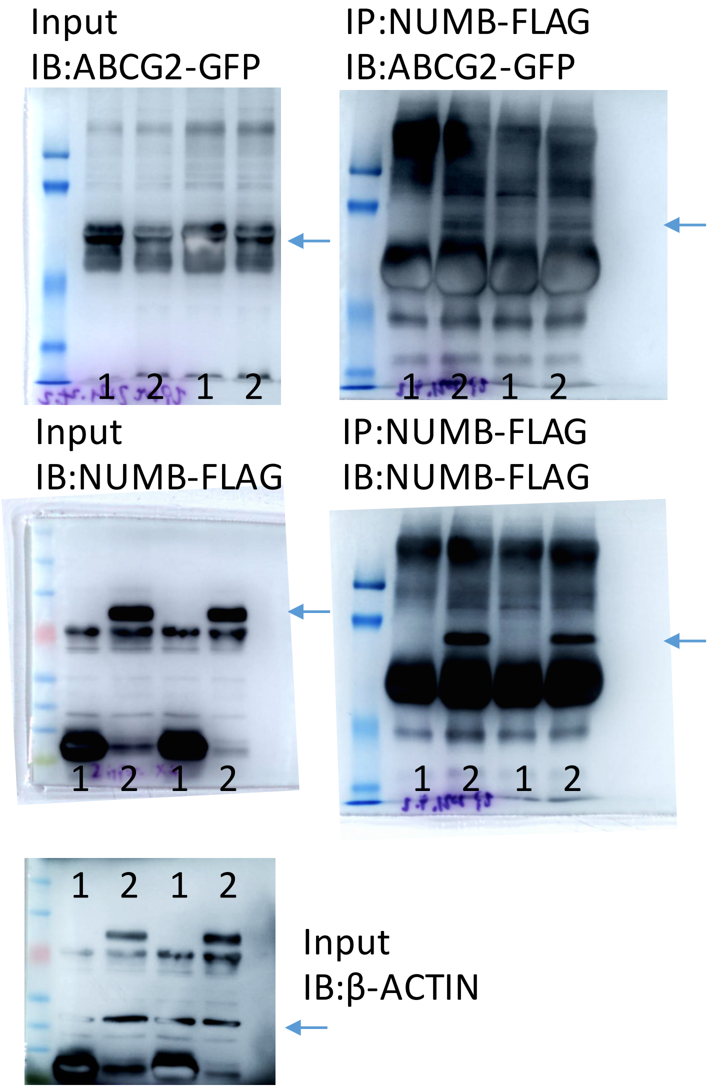

IP with FLAG antibody in HEK293 cells co-transfected with empty vector and ABCG2-GFP plasmid (negative control 1) and those co-transfected with NUMB-FLAG and ABCG2-GFP plasmids (2)

Raw data for Figure 3e

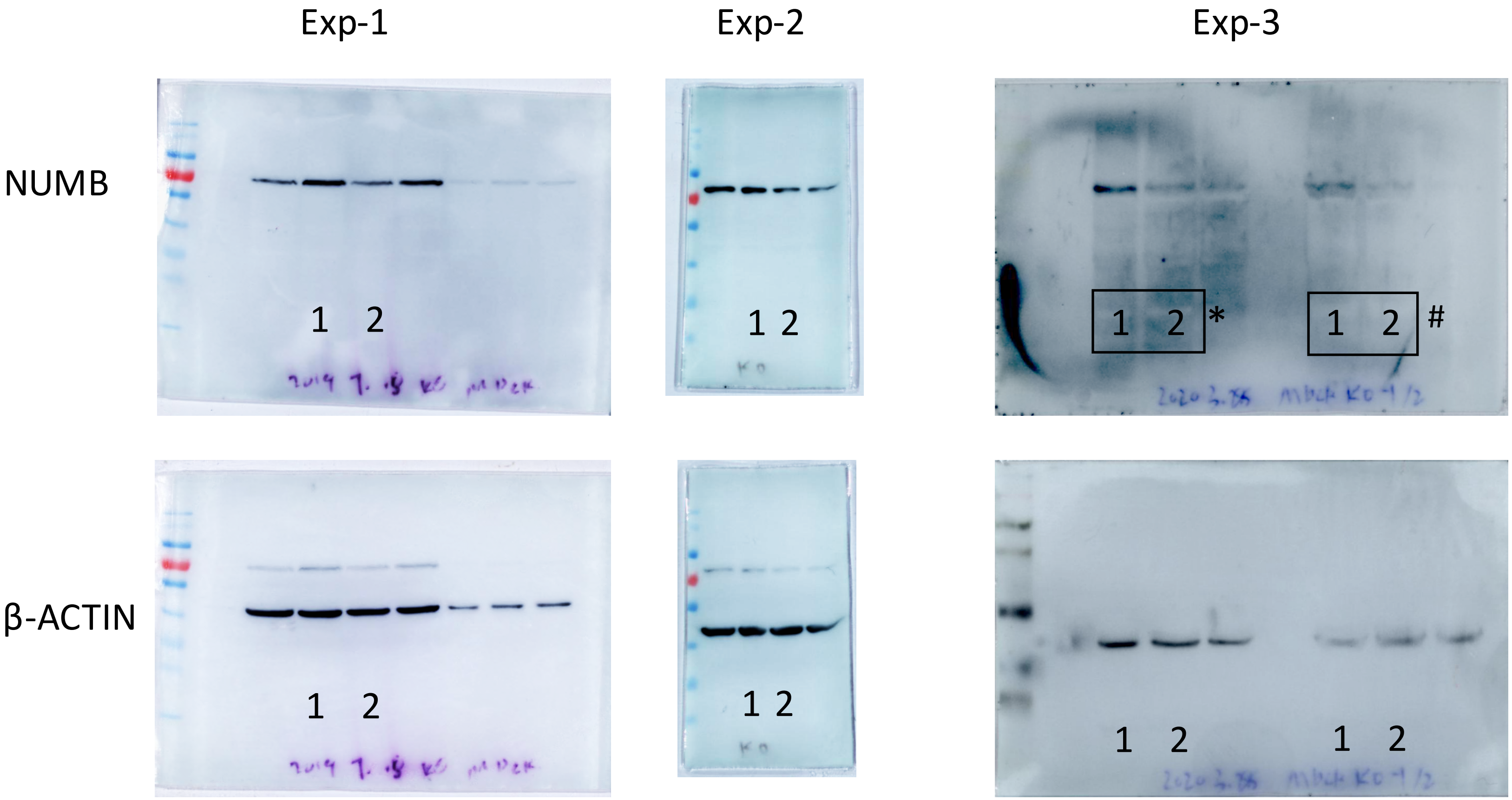

1: Vector 2: KO * and # indicate distinct samples

Raw data for Figure 4a

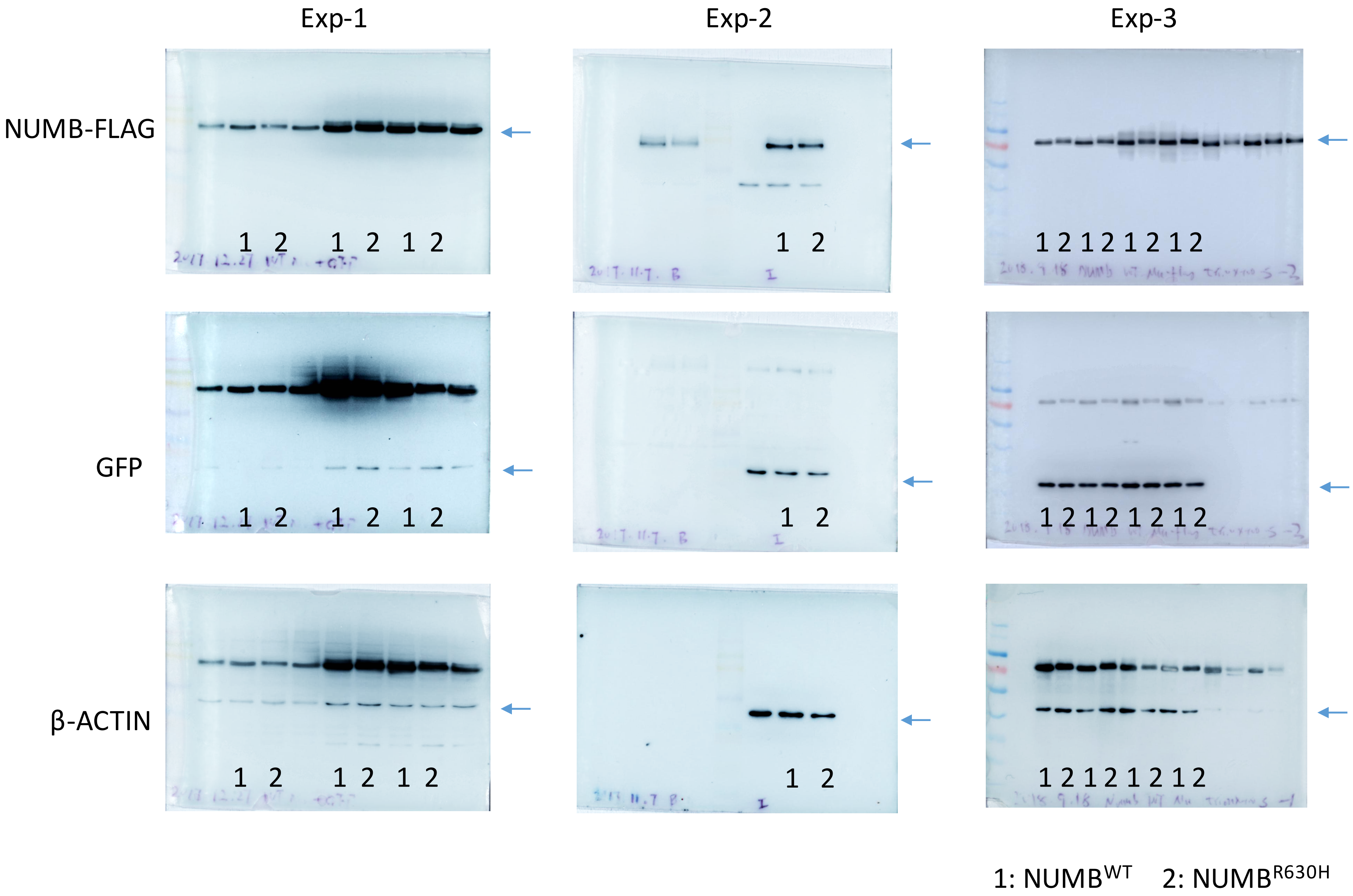

Raw data for Figure 4 f

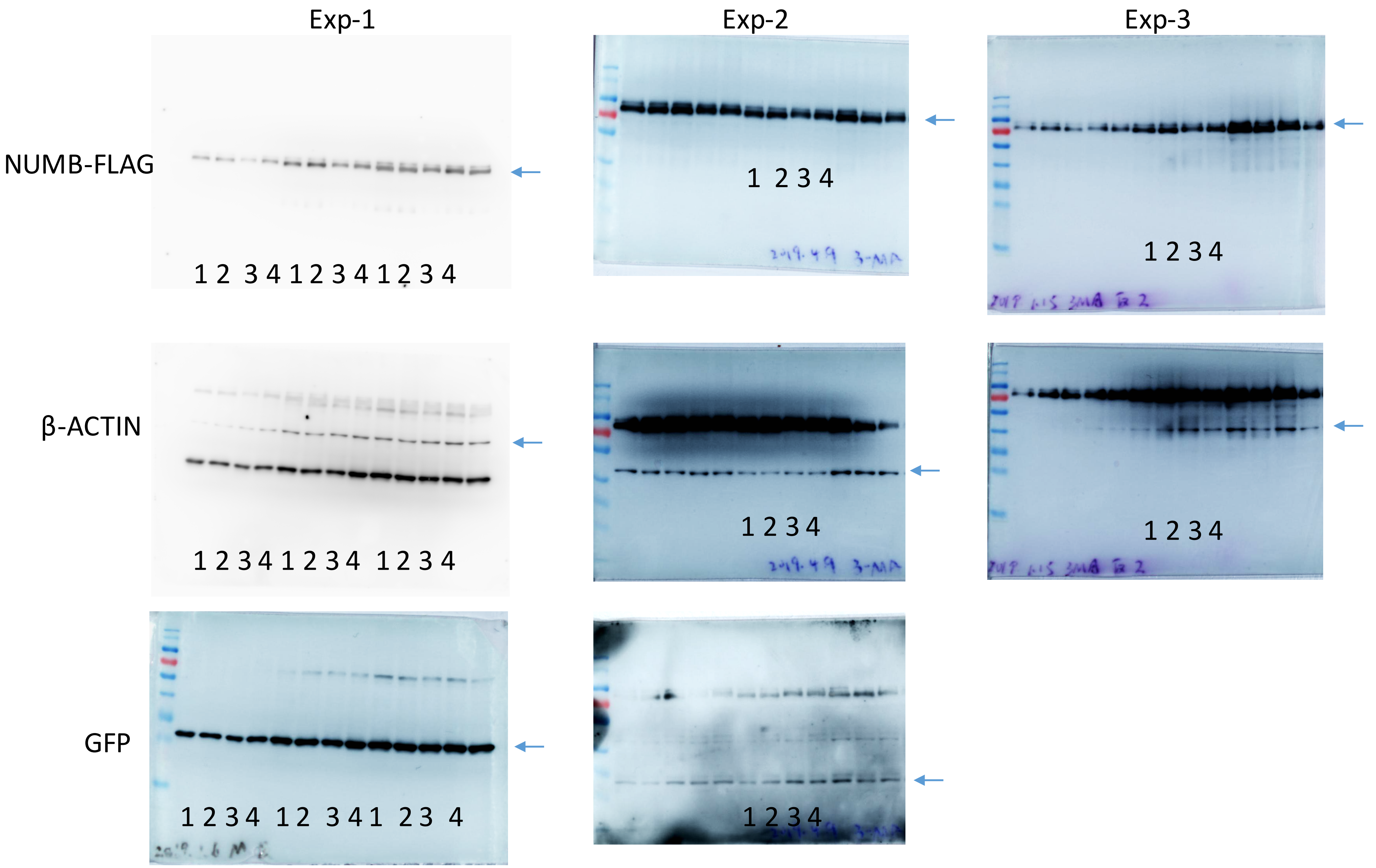

1: +WT+DMSO 2: +WT+3-MA 3: +R630H+DMSO 4:+R630H+3-MA

Raw data for Figure 4g

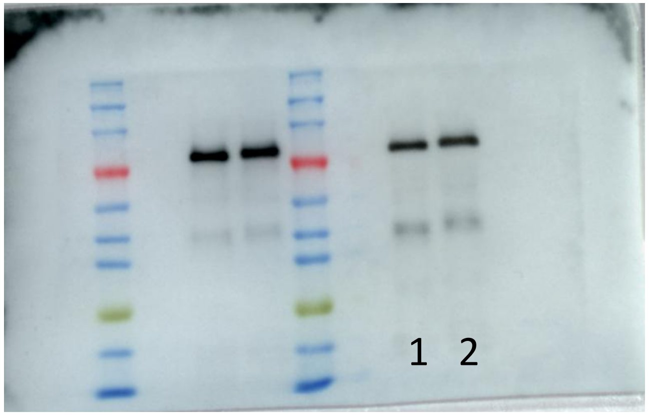

1: NUMB^{WT} 2: NUMB^{R630H}

Raw data for Figure 4 n

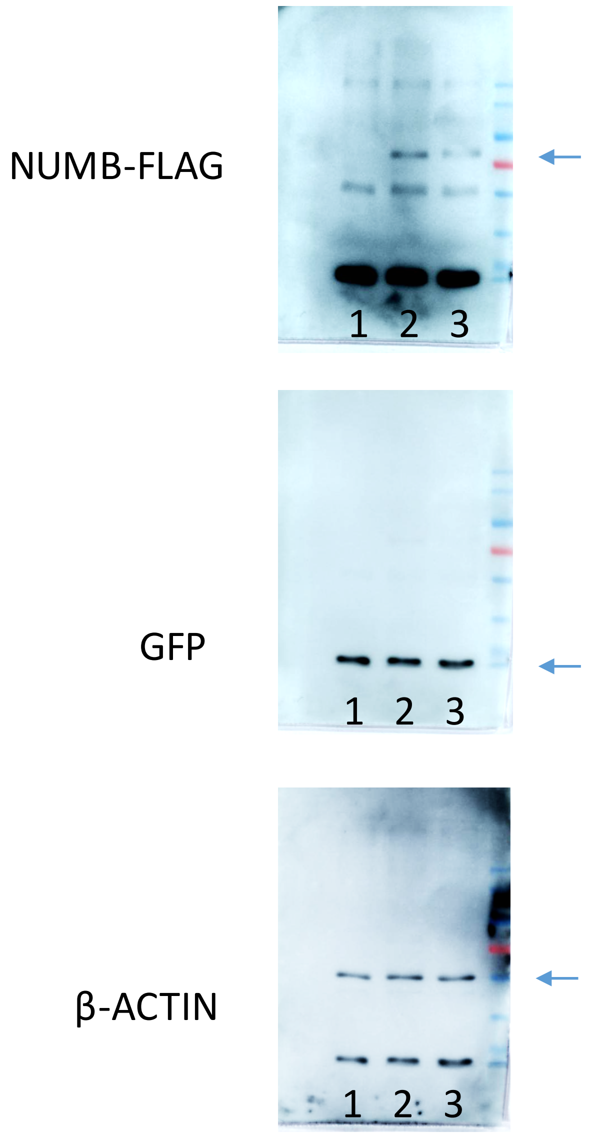

1: Vector 2: WT 3: R630H

Raw data for Extended data Figure 3b

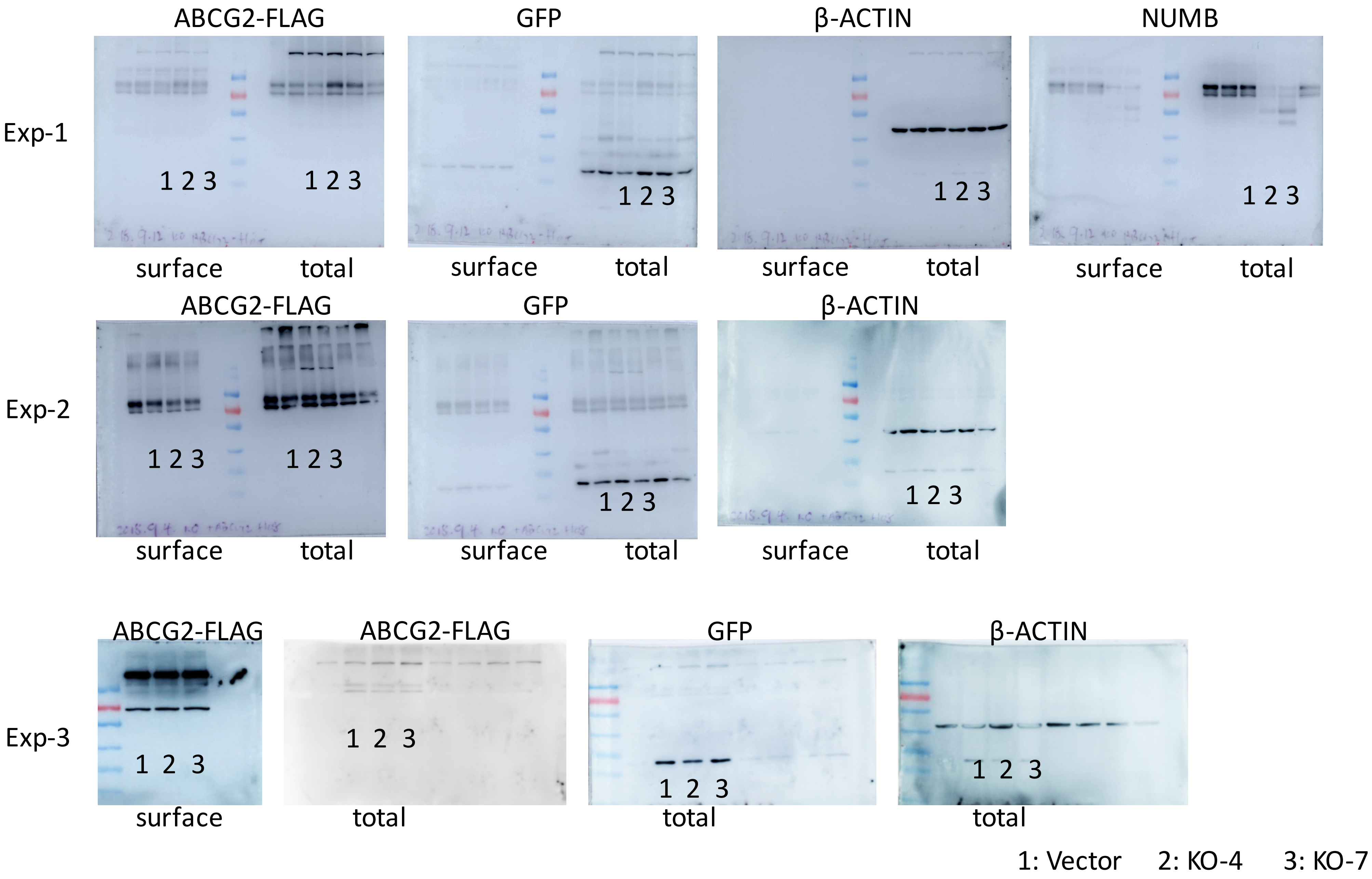

Supplement: Supplementary file 2 — Raw data [file 41421_2024_708_MOESM2_ESM.pdf]
